# “Should I stay or should I go”—a kinase delays escape of *Candida glabrata* from macrophages

**DOI:** 10.1128/mbio.03885-25

**Published:** 2026-02-20

**Authors:** Theresa Lange, Colin Clairet, Luisa Fischer, Raghav Vij, Johannes Sonnberger, Julia Mantke, Nadja Jablonowski, Eric Seemann, Britta Qualmann, Christophe d’Enfert, Lydia Kasper, Bernhard Hube, Sascha Brunke

**Affiliations:** 1 Department of Microbial Pathogenicity Mechanisms, Leibniz Institute for Natural Product Research and Infection Biology, Hans Knoell Institute 28406, Jena, Germany; 2 Unité Biologie et Pathogénicité Fongiques, Institut Pasteur, Université Paris Cité, INRAE USC2019 652302 , Paris, France; 3 Institute for Biochemistry I, Jena University Hospital, Friedrich Schiller University Jena 9378, Jena, Germany; 4 Institute of Novel and Emerging Infectious Diseases, Friedrich-Loeffler-Institut, Greifswald, Germany; 5 Institute of Microbiology, Friedrich Schiller University 163335, Jena, Germany; 6 Cluster of Excellence Balance of the Microverse, Jena, Germany; Yonsei University, Seoul, Republic of Korea

**Keywords:** host-pathogen interactions, antifungal resistance, *petite*, *Candida glabrata*, macrophages, trojan horse, intracellular fungi, immune evasion

## Abstract

*Candida glabrata* is an opportunistic fungal pathogen that causes both superficial and systemic infections in humans, accounting for 15%–25% of invasive candidiasis cases. Macrophages play a crucial role in antifungal immunity by internalizing *C. glabrata*; however, the fungus has evolved strategies to survive and even proliferate within phagosomes. It has been suggested that *C. glabrata* may utilize its life within macrophages to evade immune detection and disseminate throughout the body. We observed that, compared to fungi like *C. albicans*, *C. glabrata* only slowly escapes from macrophages, with host cells bursting after 2–3 days. This delay is fungal-driven rather than host-induced and is not solely due to replication in the yeast form *per se*. We identified protein kinases involved in exit timing, especially the Ksp1 kinase, the deletion of which accelerates macrophage cell lysis. Its loss increases mitophagy and the formation of *petites,* a respiration-deficient phenotype associated with resistance toward antifungals and, more importantly, to phagocytic killing. Moreover, deletion of *KSP1* enhances resistance to multiple antifungals, suggesting that this kinase may be at the core of a broader cross-adaptive survival strategy by *C. glabrata*. Collectively, our findings indicate that *C. glabrata* may actively prolong its intramacrophagal phase, which could contribute to immune evasion, antifungal resistance, and potential recurrence of infection. Moreover, these results reinforce the notion of a critical role of *petite* formation in persistent and recurrent infections. They also show the need to adapt clinical diagnostics and therapy to detect and manage these respiration-deficient variants.

**IMPORTANCE:**

*Candida glabrata* is a major cause of invasive candidiasis and is difficult to treat due to its intrinsic resistance to antifungal drugs and its ability to survive inside host immune cells. How this pathogen regulates its intracellular lifestyle and exit from macrophages remains poorly understood. We show that *C. glabrata* actively modulates its interaction with macrophages through the protein kinase Ksp1, which regulates mitochondrial dysfunction and the formation of respiration-deficient cells. These variants display enhanced resistance to two antifungal drugs and killing by phagocytes. Our findings suggest that prolonging the intramacrophage phase and generating stress-resistant variants are key components of *C. glabrata*’s survival strategy. Recognizing these processes has important implications for clinical diagnostics and the management of persistent and recurrent fungal infections.

## INTRODUCTION

Several *Candida* species are opportunistic pathogens that can cause superficial and even severe systemic infections in humans, affecting more than 150 million people each year (1, 2). Although the majority of these cases can be attributed to *Candida albicans*, there has been a notable rise in the incidence of infections by non-*albicans Candida* species (3). Among these, *C. glabrata* is the most prevalent (4, 5), accounting for 15%–25% of all invasive candidiasis cases in the United States and Europe (3, 6). This is at least partially due to *C. glabrata*’s high intrinsic levels of resistance to frequently used antifungal drugs, such as azoles, and its ability to quickly gain additional resistance (7, 8). Moreover, the rising incidence of *C. glabrata* infections is also likely the consequence of increasing numbers of immunocompromised patients and a growing elderly population (9).

During infections, *C. glabrata* faces different types of immune cells, including phagocytes of the innate immune system, which play a pivotal role in the initial defense against *Candida* infections (10). Particularly, macrophages are important for the clearance of tissue-invading fungi and to orchestrate downstream antifungal immune responses (11). Macrophages can efficiently internalize *C. glabrata* cells; however, the fungus has evolved strategies to survive and even proliferate within the phagosome (5, 12–15). In contrast to *C. albicans*, which forms filaments inside macrophages, induces pro-inflammatory responses, and escapes quickly (13, 16–18), *C. glabrata* replicates solely in the yeast morphology and can survive inside the phagocytes for several days (15). Despite its significant intracellular proliferation, *C. glabrata* does not elicit an apoptotic or pyroptotic host cell death or even substantial host cell damage like *C. albicans* (15, 19). Furthermore, intracellular *C. glabrata* cells induce only very little pro-inflammatory cytokine secretion (15). However, *C. glabrata* does trigger a significant level of GM-CSF response by macrophages both *in vitro* and *in vivo* (15, 20). One function of GM-CSF is the recruitment of monocytes and the activation of macrophages (21). Therefore, it has been postulated that *C. glabrata* may induce this cytokine specifically to attract macrophages as suitable host cells for intracellular persistence (13, 22, 23). This idea is strengthened by recent publications that show that *C. glabrata* can survive and replicate within THP1-derived macrophages for up to 5 days (24) and within primary macrophages for at least 7 days (25). It is, therefore, conceivable that *C. glabrata* attracts macrophages for intracellular persistence in a “Trojan horse” strategy to evade the immune system (26).

Persistence of bacteria such as *Mycobacterium tuberculosis* is associated with drug tolerance and resistance (27), and a similar association has been shown for fungal persistence (24, 25). In fact, *C. glabrata*’s persistence in macrophages is known to increase the emergence of echinocandin-resistant colonies, which is specifically associated with its non-proliferative stage (24). A major trigger of this resistance appears to be macrophage-induced oxidative stress, as deletion of fungal ROS-detoxifying enzymes increases the emergence of echinocandin-resistant colonies (24). Moreover, persistence of *C. glabrata* in primary macrophages can induce a so-called *petite* phenotype, which is associated with mitochondrial dysfunction and confers cross-resistance against both phagocytic killing and antifungal drugs such as azoles or echinocandins (25, 28).

The precise processes leading to intracellular persistence are unclear. For example, it may be possible that the host forces *C. glabrata* into an intracellular persistence mode; alternatively, the process could be driven by the fungus itself. If *C. glabrata* actively hijacks phagocytes *in vivo*, however, it should be able to precisely regulate the timing of its escape from the macrophage. Such a strategy would allow *C. glabrata* cells to evade the host immunity for an extended period and enable fungal cells to reach distal body parts safely. Nevertheless, the intracellular environment of a macrophage is inherently hostile and generally lacking in nutrients; therefore, *C. glabrata* must eventually escape.

In this study, we focused on investigating the mechanism by which *C. glabrata* exits macrophages, with a particular emphasis on understanding the processes involved in the delayed escape of this yeast. We identified a fungal protein kinase that plays a key role in these processes. We suggest that this kinase, Ksp1, modulates the formation of *petite* cells by regulating mitochondrial autophagy. This, in turn, affects the intracellular fungal load and thus the escape rate, as demonstrated by our observation that a higher fungal load leads to an earlier lysis of macrophages.

## RESULTS

### *Candida glabrata*’s replication as yeast cells does not *per se* delay its exit from macrophages

**Fig 1:**
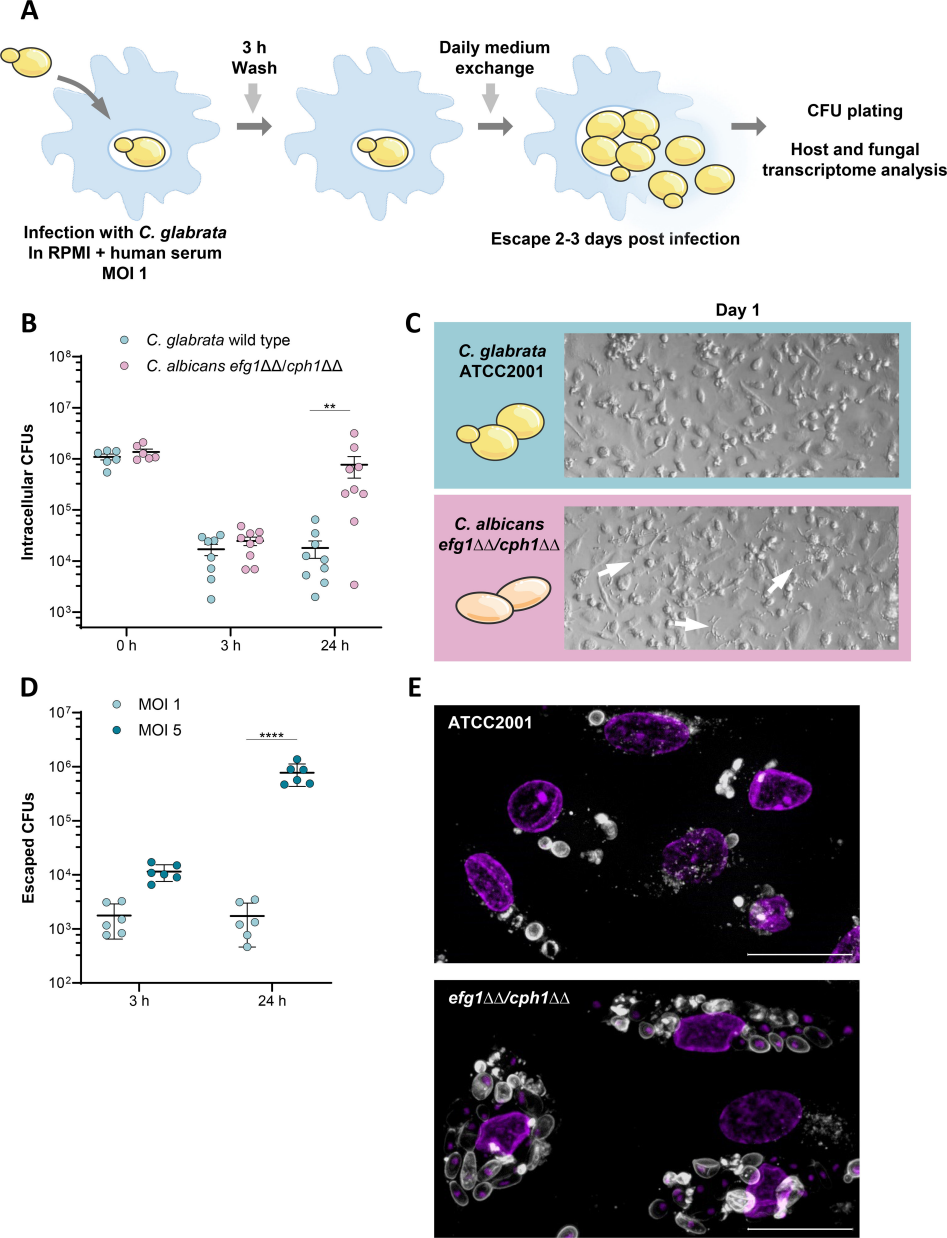
Interaction of *C. glabrata* wild type and *C. albicans* yeast-locked *efg1*ΔΔ/*cph1*ΔΔ mutant cells with macrophages. (**A**) *C. glabrata* escape model using monocyte-derived macrophages based on a previously published persistence model (25). (**B**) Intracellular survival of *C. glabrata* and the *C. albicans* yeast-locked mutant *efg1*ΔΔ/*cph1*ΔΔ in human monocyte-derived macrophages. Macrophages were lysed at the indicated time points, and intracellular yeast cells were plated. Each dot indicates one donor (*n* = 6–9 donors). Results were compared using a two-way ANOVA with Šídák’s multiple comparisons test (**, *P* < 0.01). (**C**) Representative micrograph of infected macrophages in the escape model after 1 day. Arrows indicate escaped yeast cells. (**D**) Extracellular CFUs of *C. glabrata* after infecting human monocyte-derived macrophages with different multiplicities of infection (MOIs). Each dot indicates one donor (*n* = 6 donors). Results were compared using a two-way ANOVA with Sídák’s multiple comparisons test (****, *P* < 0.0001). (**E**) Intracellular yeasts, i.e., the *C. glabrata* wild type and the yeast-locked *C. albicans* mutant *efg1*ΔΔ/*cph1*ΔΔ, were fixed 3 h after infecting human monocyte-derived macrophages (MOI 5). Fungal cells were stained with ConA-AlexaFluor647, and macrophage nuclei were labeled with DAPI. The brightness of the representative images was increased by 20%. Scale bar indicates 20 µm.

To study the mechanism and dynamics of *C. glabrata*’s escape from macrophages, we established a long-term human monocyte-derived macrophage (hMDM) interaction model (Fig. 1A) based on a previously published persistence model (25). Briefly, for this model, hMDMs are infected with *C. glabrata*, and non-phagocytosed fungal cells are removed 3 h post-infection. The intracellular survival and subsequent escape process can be studied for up to 3 days by multiple read-outs, such as colony-forming unit (CFU) measurement, transcriptomic analyses, and damage assays.

As *C. albicans* primarily relies on hypha formation to escape from macrophages (13) and escapes more rapidly than *C. glabrata* (15, 22), we hypothesized that the delayed exit of *C. glabrata* may simply be due to the lack of hypha formation. To investigate the role of morphology (i.e., the presence of filaments vs. the yeast form), we compared the dynamics of intracellular proliferation between the *C. glabrata* wild-type cells and cells of the yeast-locked *C. albicans* mutant, *efg1*ΔΔ/*cph1*ΔΔ (29) (Fig. 1B and C). Surprisingly, the yeast-locked *C. albicans* mutant proliferated significantly faster inside macrophages than *C. glabrata* (Fig. 1B and E) and, thus, escaped after a mere 24 h of infection (Fig. 1C; Fig. S1). In contrast, *C. glabrata* exited the phagocytes after 2–3 days, when macrophages containing high numbers of replicating *C. glabrata* cells burst (Fig. S1), as published previously (15). This demonstrates that fungal replication in the yeast form *per se* does not mediate the delayed exit by *C. glabrata*.

The higher intracellular fungal load observed for the yeast-locked *C. albicans* mutant indicates that the fungal burden itself might play a role in the timing of the macrophage rupture. To test this further, we infected hMDMs with *C. glabrata* at different multiplicities of infection and later quantified the escaped CFUs (Fig. 1D). A higher initial fungal load resulted in a significant increase in extracellular CFU numbers, indicating an earlier exit. This suggests rupture of the macrophages by the fungal burden itself as one mechanism by which *C. glabrata*, and potentially also the yeast-locked *C. albicans* mutant, can escape.

### Species-independent transcriptional responses of macrophages to internalized yeast cells

As the fungal morphology does not seem to be the primary factor in delaying *C. glabrata*’s exit, we postulated that macrophages might respond differently to yeast-locked *C. albicans* mutant and *C. glabrata* wild-type yeast cells despite their comparable morphologies. To elucidate whether macrophages actively steer *C. glabrata* onto a delayed exit trajectory, we performed transcriptomic analyses on hMDMs following the phagocytosis of *C. glabrata* or *C. albicans efg1*ΔΔ/*cph1*ΔΔ cells. To this end, we adapted the established exit model and obtained host samples at eight distinct time points over the course of a 3-day infection.

**Fig 2:**
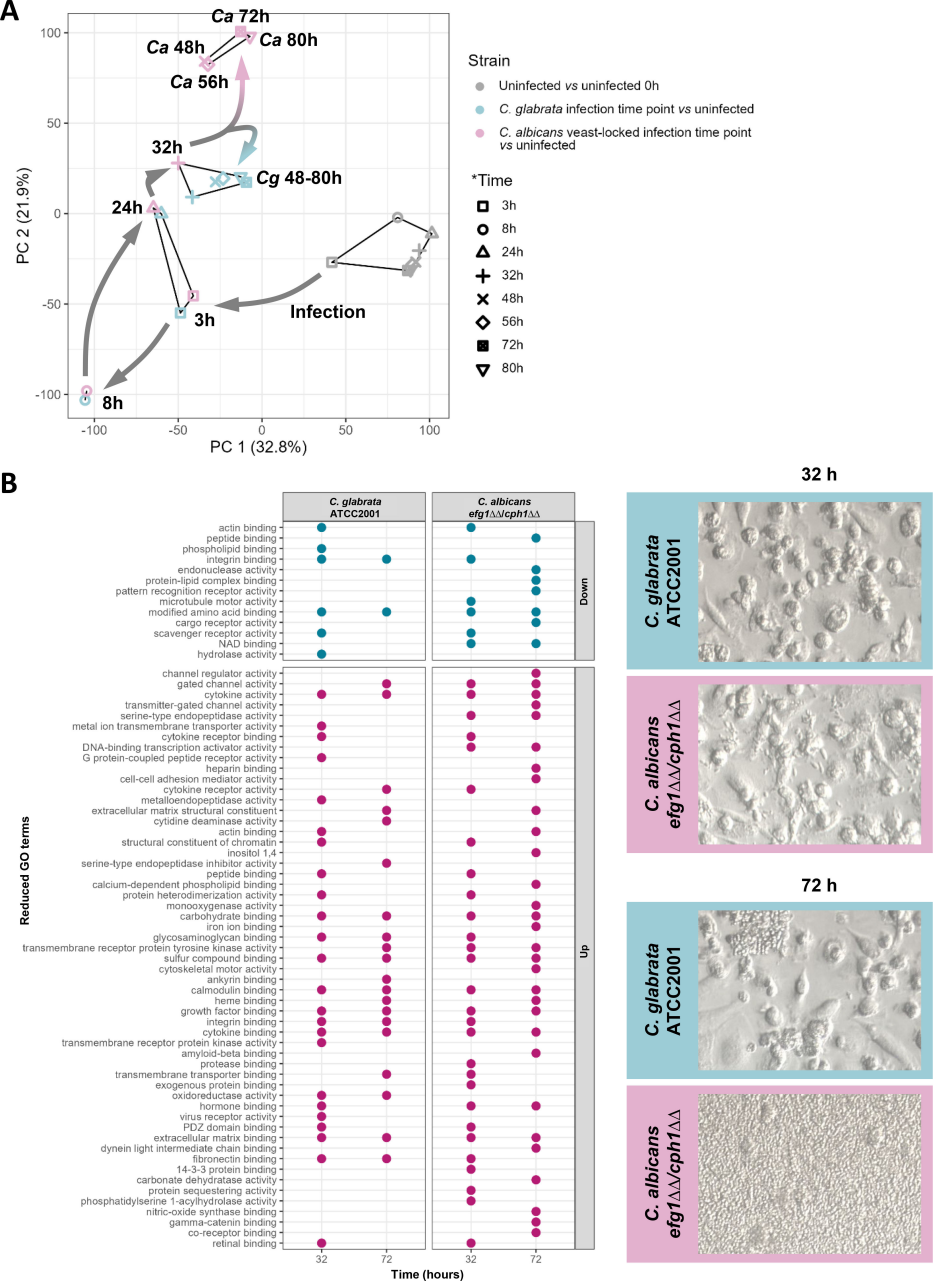
Macrophage transcriptional response to *C. glabrata* wild type and the yeast-locked *C. albicans* mutant *efg1*ΔΔ/*cph1*ΔΔ. (**A**) PCA of all sampled time points during the yeast-macrophage interaction in the escape model. Uninfected samples are depicted in gray and are shown relative to the uninfected 0 h time point. *C. glabrata*- (blue) or *efg1*ΔΔ/*cph1*ΔΔ- (pink) infected macrophage samples are shown relative to the respective uninfected sample at the indicated time point (*n* = 3 donors). (**B**) Reduced gene ontology (GO) term analysis of macrophages’ transcriptomes relative to the uninfected samples at the same time points. Transcriptomic analyses were performed with macrophages from three donors, and the mean was used for further analyses.

A principal component analysis (PCA; Fig. 2A) showed that uninfected hMDMs have a comparable transcriptome across all three different blood donors throughout the entire infection period, setting a solid baseline for our comparisons. Upon infection, the macrophage transcriptome underwent a shift along the PC1 axis at 3 h for both fungal species, *C. glabrata* and the yeast-locked *C. albicans* mutant. This shift was even more pronounced at the 8 h time point. At both time points, however, the *C. glabrata*- and *C. albicans*-induced macrophage response was strikingly similar, with samples from both always clustering together in the PCA plot. This also applied to the 24 h and 32 h time points, where a shift along the PC2 axis is evident for both samples. This indicates that the early transcriptomic response of the macrophages is largely independent of the fungal species.

After 32 h of infection, the phagocyte response to *C. glabrata* started to diverge from the response to the yeast-locked *C. albicans* mutant. For the *C. glabrata* infection, the macrophage transcriptome of the later time points (up until 80 h post infection) clustered together with this 32 h time point, indicating minimal or no further changes in the response. The response to yeast-locked *C. albicans* cells exhibited a further shift along the PC2 axis, yet all subsequent time points cluster together.

Overall, we did not observe a difference in the early macrophage response to both yeast cells, which would be the expected time at which a divergence should occur, potentially steering one toward a postponed exit. Thus, we conclude that the macrophages do not play a significant role in slowing down the exit event, which, in turn, suggests that *C. glabrata*’s delayed escape is likely fungal-driven.

### The late macrophage response differs slightly between yeast cells of both species

Given the disparate late macrophage transcriptomic responses to the two tested yeast cell strains, we conducted a reduced Gene Ontology term analysis for the differentially regulated genes at two time points, 32 h and 72 h post-infection (Fig. 2B). At the earlier of these time points, *C. glabrata* replicates intracellularly, whereas the yeast-locked *C. albicans* mutant can escape from the phagocytes. At the later time point, *C. glabrata* exits the macrophage, while the *C. albicans* mutant already continues replicating in the medium, resulting in overgrowth of the host cells. As expected from the PCA plot findings, the enriched GO terms for upregulated and downregulated processes were similar for infections with both fungal species at the early 32 h time point (Fig. 2B), with few exceptions. Macrophages showed a specific upregulation of receptor- and signaling-associated processes in response to infection with the *C. albicans* mutant. Nevertheless, the *C. glabrata*-infected host cells exhibited a GO term enrichment of these signaling processes at the later 72 h time point, indicating a slightly delayed (pro-) inflammatory response toward *C. glabrata*.

To further investigate the macrophage inflammatory responses to either the *C. glabrata* or *C. albicans efg1*ΔΔ/*cph1*ΔΔ infection, we specifically compared infection-associated genes encoding chemokines and cytokines. Supporting the PCA plot, the transcriptional responses were generally very similar to both infecting species (Fig. S2). Among the most strongly upregulated genes in the first hours, we found, as expected, those coding for the lysosomal marker LAMP-3 and for pro-inflammatory cytokines such as IL-1α and IL-1β or IL-6. Especially at time points later than 8 h after uptake of the fungi, we saw a strong upregulation of CC chemokine genes, especially *CCL1*, *CCL17*, and *CCL19*. This profile of upregulated chemokines suggests a persistent pro-inflammatory signaling and an attraction especially of Th17-type cells that play a central role in fungal immunity (30).

With a few subtle exceptions, the pattern of these responses was nearly identical during the first 56 h of infection (in which a significant fraction of macrophages was still alive). Only when specifically looking for differences between the *C. glabrata* and *C. albicans efg1*ΔΔ/*cph1*ΔΔ-infected macrophages, did we observe a very strong upregulation of genes involved in hypoxia response in the *efg1*ΔΔ/*cph1*ΔΔ-infected macrophages (Fig. S2). This response included *HILPDA* (coding for a hypoxia-inducible lipid droplet-associated protein), *EGLN3* (hypoxia-inducible regulator of HIF-1 and others), and genes for members of the heat-shock factor 70 family (*HSPA1A*, *HSPA1B*, *HSPA6*). The hypoxia-type reaction was, however, either not activated in macrophages infected with *C. glabrata* or, interestingly, only present in the early time points (3 h and 8 h) with a later drop in transcript levels. This is supported by a gene set enrichment analysis performed on KEGG pathways (31), which shows the HIF-1 (hypoxia-induced factor) signaling pathway to be enriched for lower transcript levels after uptake of *C. glabrata* compared to *efg1*ΔΔ/*cph1*ΔΔ.

### The *C. glabrata* kinase Ksp1 is involved in the interaction with macrophages

**Fig 3:**
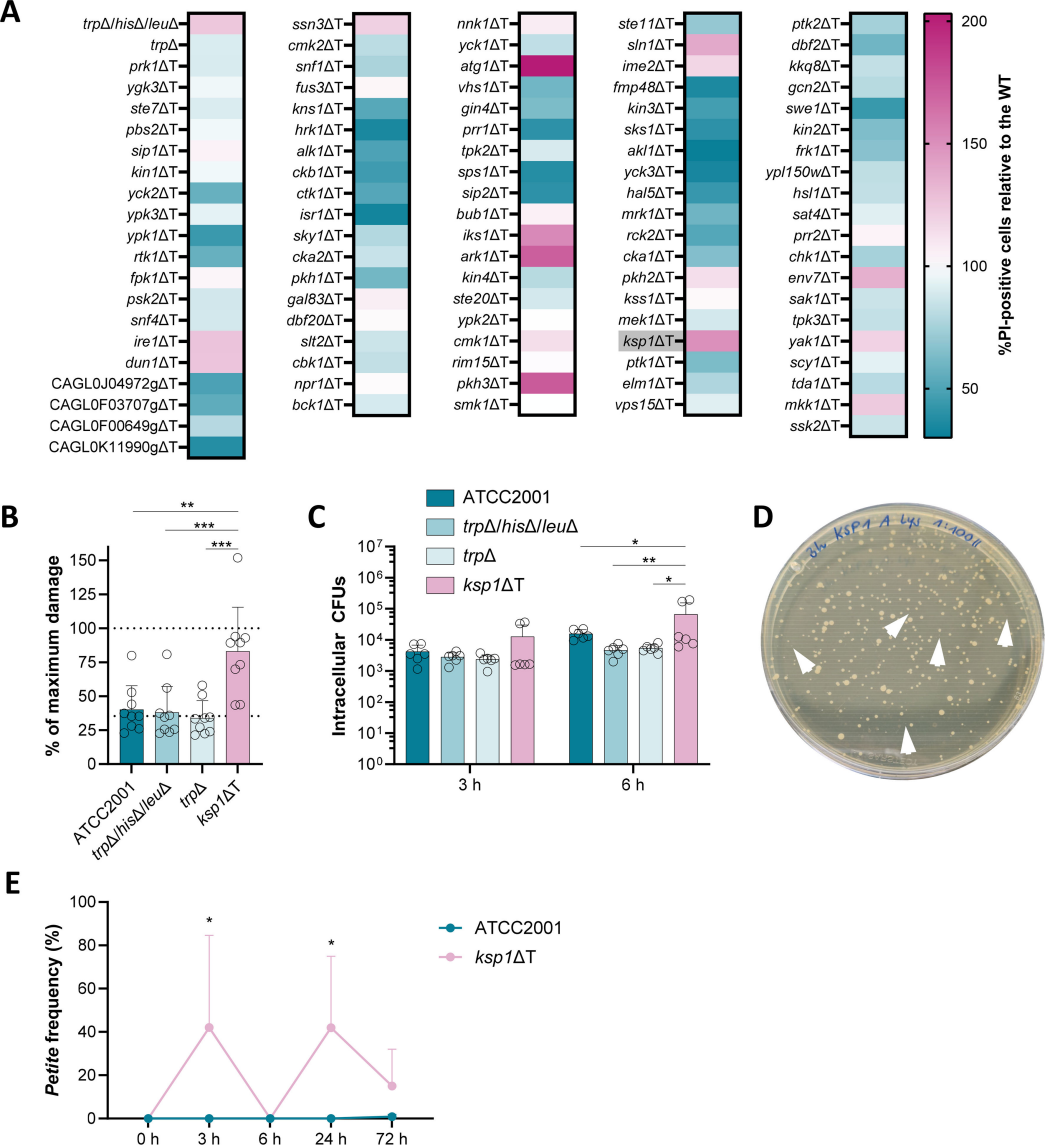
Screening of a *C. glabrata* kinase mutant library and characterization of a mutant lacking the Ksp1 kinase in macrophages. (**A**) *C. glabrata* kinase mutant library screening using propidium iodide staining of murine J774A.1 macrophage-like cells 24 h post-infection. Data are shown relative to *C. glabrata* wild-type strain ATCC2001 (*n* = 3). (**B**) Damage induction to primary human macrophages measured by quantification of lactate dehydrogenase release caused by three parental *C. glabrata* strains and the *ksp1*ΔT kinase mutant 24 h post infection (*n* = 6 donors). The lower dashed line indicates the LDH release background measured with an uninfected control. Statistical significance was calculated using a one-way ANOVA with Tukey’s multiple comparisons test (**, *P* < 0.01; ***, *P* < 0.001). (**C**) Survival of intracellular *C. glabrata* parental strains and the *ksp1*ΔT kinase mutant in primary human macrophages was determined by lysing and plating intracellular CFUs at the indicated time points (*n* = 6 donors). Statistical significance was calculated using a two-way ANOVA with Tukey’s multiple comparisons test (*, *P* < 0.05; **, *P* < 0.01). (**D**) Representative picture of an agar plate with the intracellular CFUs of *ksp1*ΔT 3 h after infection of macrophages. Arrows indicate examples for small colony variants. (**E**) Percentage of *petite* CFUs relative to the overall intracellular CFU numbers throughout the 3-day infection course in hMDMs (*n* = 5 donors). Statistical significance was calculated using a two-way ANOVA with Šídák’s multiple comparisons test (*, *P* < 0.05). Asterisks indicate significance compared to the ATCC2001 strain.

Given that *C. glabrata*’s delay in exit from the macrophage seemed to be driven by the fungus itself, we sought to identify fungal elements involved in facilitating the persistence phase, or those that drive escape and regulate the timepoint when *C. glabrata* exits the host cell. To this end, we turned to kinases because of their well-established role as mediators of signaling cascades. We screened a *C. glabrata* non-essential kinase mutant library in J774A.1 macrophage-like cells, using propidium iodide staining as a marker for host cell lysis (Fig. 3A). We identified several fungal kinases that seem to play a role in the cell lysis of macrophages. Of the 96 mutants tested, 34 induce 75% or less of ATCC2001 (wild type)-like host cell lysis levels (indicated in dark blue in the figure), suggesting that the corresponding kinases may be factors that contribute to the exit event. Only seven kinase mutants exhibited an augmented host cell lysis, reaching 135% or more of the wild-type level (indicated in dark pink). These kinases may play a role in delaying the escape process and maintaining the intramacrophagal localization of *C. glabrata*. This includes the mutant lacking *KSP1* (*Saccharomyces cerevisiae* orthologs have a role in TOR signaling, negative regulation of macroautophagy, regulation of translation in response to stress [32]), *PKH3* (orthologs have a role in the cell wall integrity MAPK cascade [32]), *ARK1* (potential role in actin filament organization, regulation of clathrin-dependent endocytosis and actin cortical patch localization [32]), *IKS1* (unknown role [32]), *ATG1* (required for induction of autophagy under nitrogen starvation and oxidative stress [33]), *SLN1* (involved in two-component signaling pathway [34]), or *ENV7* (orthologs have a role in regulation of vacuole fusion, non-autophagic, vacuolar protein processing [32]).

We proceeded to test the seven strongly lysing kinase mutants individually in primary human macrophages (Fig. 3B through D; Fig. S3). In this model, we evaluated their capacity to induce damage 24 h post infection, as well as their ability to survive and proliferate within macrophages (at 3 and 6 h post infection). In contrast to the results obtained from the screening in J774A.1 macrophage-like cells, only three mutants induced a significantly higher level of damage to the primary macrophages than the wild type (Fig. 3B; Fig. S3A). The genes deleted in these mutants were *KSP1*, *ENV7*, and *ATG1*. Notably, only the deletion of *KSP1* also resulted in a significantly increased survival and replication of *C. glabrata* within primary macrophages at 3 h and 6 h post infection (Fig. 3C; Fig. S3C). The other tested mutants did not reach the intracellular CFU numbers of the wild type. To better differentiate the deletion mutant in the library (which was created using a tryptophan prototrophy marker) and a later independent deletion mutant (created with the dominant NAT resistance marker), we named the former mutant *ksp1*ΔT throughout the manuscript (and the latter *ksp1*ΔN; see below). Since the *ksp1*ΔT mutant induced the greatest level of macrophage lysis and showed an enhanced intracellular proliferation, we proceeded to investigate the mechanisms through which the Ksp1 kinase is involved in the *C. glabrata*-macrophage interaction.

### Growth of the *ksp1*ΔT mutant in macrophages leads to a *petite*-like phenotype

In the course of evaluating the intracellular survival and replication rate of this kinase mutant, we observed small colony variants on agar plates after the retrieval of the *ksp1*ΔT mutant from macrophages (Fig. 3D). This phenotype resembled previously described *petites*, a small colony variant, which is associated with mitochondrial dysfunction and confers cross-resistance to phagocytic killing and fluconazole (25). In this earlier study, *petites* were shown to emerge mostly after 1 day of macrophage interaction. Therefore, we examined the emergence rate of *petites* in *ksp1*ΔT-infected macrophages (Fig. 3E) and confirmed the highest frequency of occurrence after 3 h and 24 h post-infection of primary macrophages. At 72 h post-infection, a minority of cells were *petites*, whereas no *petites* were observed at 6 h post-infection. The wild type showed a frequency of 0%–1% at all time points, as reported by a previous study (25).

### The *C. glabrata ksp1*ΔT mutant shows only some of the *petite* hallmarks

**Fig 4:**
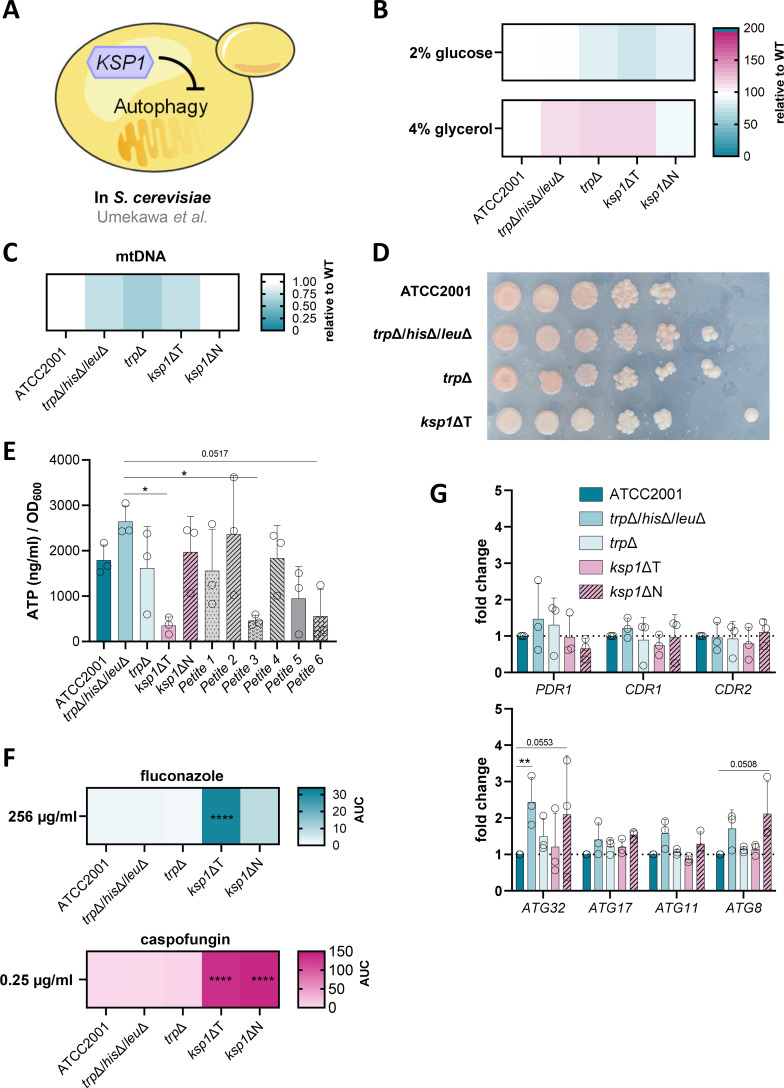
Characterization of the *ksp1*Δ mutant regarding the *C. glabrata petite* hallmarks. (**A**) Role of the Ksp1 kinase in *Saccharomyces cerevisiae* based on reference 35. One of Ksp1’s functions, among others, is the negative regulation of macroautophagy via the TORC1 pathway, which would affect mitochondrial functions as well. (**B**) Growth of the parental *C. glabrata* strains, the *ksp1*∆T kinase mutant from the kinase library, and the independently constructed *ksp1*∆N mutant in SD media supplemented with either 2% glucose or 4% glycerol as an alternative carbon source at 37°C for 4 days. Growth is shown as the mean of the area under the curve (*n* = 3). (**C**) Presence of mitochondrial DNA in the parental *C. glabrata* strains and both *ksp1*∆ kinase mutants was quantified from overnight cultures via PCR of the mitochondria-associated gene *COX3* relative to *ACT1* and to the levels in the wild type ATCC2001 (*n* = 3). (**D**) Mitochondrial function was determined by dropping serial dilutions of the parental *C. glabrata* strains and *ksp1*∆ kinase mutants on SD+4% glycerol agar containing 0.02% tetrazolium chloride, indicating functional mitochondria by dye reduction. Plates were grown at 37°C for up to 4 days (*n* = 2). (**E**) ATP concentration was measured from log-phase cultures of the indicated strains grown in YPD at 37°C, 180 rpm, and is depicted relative to the measured OD_600 nm_ (*n* = 3). Statistical significance was calculated using a one-way ANOVA with Tukey’s multiple comparisons test (*, *P* < 0.05). (**F**) Antifungal susceptibility of the parental *C. glabrata* strains and both *ksp1*∆ kinase mutants measured by performing growth curves in YPD supplemented with the indicated antifungal concentration at 37°C for 3 days. Growth is shown as the mean area under the curve (*n* = 3). Statistical significance was calculated using a one-way ANOVA with Tukey’s multiple comparisons test (****, *P* < 0.0001). Asterisks indicate significance compared to the corresponding ATCC2001 strains. (**G**) Expression of efflux pump-related (top) and autophagy-related (bottom) genes of the parental *C. glabrata* strains and both *ksp1*∆ kinase mutants as determined by qPCR. Gene expression is shown as fold change relative to *ACT1* and ATCC2001 as the corresponding wild type. The dashed line indicates no change (*n* = 3). Statistical significance was calculated using a two-way ANOVA with Dunnett’s multiple comparisons test (**, *P* < 0.01).

At this point, it was unclear whether the differences in the macrophage interactions between mutant and wild type were due to the deletion of *KSP1* itself or were rather induced by the *petites* that formed later due to this deletion. Ksp1 has yet to be characterized in *C. glabrata*; however, it is known that the Ksp1 kinase negatively regulates autophagy processes in *S. cerevisiae*, including mitophagy (Fig. 4A) (35). We hypothesized that this is also the case in *C. glabrata*, which would ultimately result in a loss of mitochondrial function and, thus, the formation of *petites*. A population of *C. glabrata ksp1*ΔT mutants, as a mixture of *petites* and *grandes* with a high propensity to switch toward the former, may therefore exhibit certain *petite* characteristics in standard assays for the *petite* phenotype (25, 28, 36). The key hallmarks of *C. glabrata petites* have been established previously (25). These include slow growth and appearance as small colonies (as observed in Fig. 3D), no growth in non-fermentable carbon sources, the loss of mitochondrial DNA (mtDNA) and function, the resistance to certain antifungal drugs, and the upregulation of efflux pump genes (25, 28).

Growth curve experiments were performed in minimal medium containing either 2% glucose as the preferred carbon source or 4% glycerol as an alternative non-fermentable carbon source. We included the three *C. glabrata* background strains as isogenic controls. As expected, all wild type strains were able to grow in the presence of 2% glucose (Fig. 4B). However, the *ksp1*ΔT strain showed wild type-like growth in the non-fermentable carbon source glycerol, which is in contrast to the previously described *petite* phenotype (25) and suggests that it retained some mitochondrial functionality. The function of Ksp1 is related to starvation responses, and it is possible that the new genomic location of the *TRP1* gene as a deletion marker influences its expression. We therefore also created a new, independent tryptophan-prototrophic mutant using a nourseothricin resistance cassette to replace *KSP1*. This *ksp1*∆N mutant did indeed show reduced growth on glycerol (Fig. 4B), supporting our suggested link of Ksp1 to nutrient homeostasis.

Given that the *petite* phenotype of *C. glabrata* is associated with the (transient) loss of mitochondrial DNA and function (25, 28), mtDNA was quantified by measuring the presence of the mitochondria-encoded gene *COX3* via qPCR in the *ksp1*ΔT mutant (Fig. 4C). Notably, only low levels of the *COX3* gene were detectable in *ksp1*ΔT, which is consistent with the *petite* phenotype. However, reduced levels of *COX3* were also observed in the two background control strains, *trp*∆/*his*∆/*leu*∆ and *trp*∆, but not in the *ksp1*ΔN strain. This leads to the assumption that there is some loss of mitochondrial DNA in these strains, which, however, is not due to the deletion of *KSP1*.

An uncoupling of the electron transport chain would also result in mitochondrial dysfunction, despite the presence of mitochondrial DNA. Hence, we investigated the mitochondrial reductive power by performing drop tests on different types of agar containing tetrazolium chloride (TTC) (Fig. 4D; Fig. S4A and B). Only mitochondria-proficient cells are capable of reducing TTC, which results in the formation of red colonies (25). On YPD-TTC agar, the *ksp1*ΔT mutant formed red colonies, indicating functional mitochondria, whereas the isogenic wild type strains grew as white colonies (Fig. S4B). The latter strains began to form red colonies after a prolonged incubation time at 37°C, as they likely switched from glucose fermentation to respiratory chain activation. To circumvent that effect, the drop tests were repeated on minimal medium containing a non-fermentable carbon source, namely glycerol, allowing the immediate reduction of TTC. Indeed, *ksp1*ΔT and *ksp1*ΔN appeared as white colonies, indicating a potential mitochondrial dysfunction, whereas the wild type changed its color to a light red (Fig. 4D; Fig. S4A and B). To prove the mitochondrial dysfunction further, ATP concentrations were determined as a measure of TCA cycle and oxidative phosphorylation functions (Fig. 4E). In fact, the *ksp1*ΔT mutant showed a fivefold lower intracellular ATP level compared to the wild type, supporting the notion of a reduction in mitochondrial functionality. However, ATP levels were similar to the wild type for the *ksp1*ΔN mutant and for several established *petites* (Fig. 4E), suggesting that this test is not necessarily conclusive on its own for detecting mitochondrial dysfunctions (37).

Furthermore, transmission electron microscopy (TEM) pictures of the *ksp1*ΔT mutant revealed smaller mitochondria compared to the *C. glabrata* wild type (Fig. S5B), which could be the cause of the reduced mitochondrial activity. Finally, MitoTracker staining confirmed that the *ksp1*ΔT mutant has low levels of active mitochondria (Fig. S5A), similar to the previously described mitochondria-deficient *mip1*∆ mutant (25).

Formation of *petites*, moreover, can result in resistance to echinocandin and azole antifungals (25, 28). To test the antifungal susceptibility of the *ksp1*ΔT mutant, growth curve experiments were performed in media containing either fluconazole or caspofungin (Fig. 4F). Notably, the *ksp1*∆T mutant seemed to be the only strain growing at the tested fluconazole concentration, with only the *ksp1*∆N also showing a slight, but not statistically significant improvement in growth compared to the wild type. The *ksp1*∆T mutant furthermore grew significantly better in the presence of caspofungin compared to its background strain, but here we saw the same improvement also for *ksp1*∆N, suggesting that deletion of *KSP1* leads to an increased propensity for *petite*-like phenomena. Reduced susceptibility to azoles is frequently driven by a *PDR1*-dependent upregulation of the efflux pump-encoding genes *CDR1* and *CDR2* (38), which has also been observed in *petites* (25). To investigate whether this phenomenon occurs in the *ksp1*ΔT mutant, the expression of the efflux pump genes was quantified via RT-qPCR (Fig. 4G). All tested strains showed transcript levels comparable to the wild type, indicating that the antifungal resistance in this mutant is due to *PDR1*-independent pathways.

Given that the *KSP1* deletion might result in a higher *petite* formation rate due to an increase in autophagy, we analyzed the gene expression levels of autophagy-associated genes via RT-qPCR (Fig. 4G). The following genes were included in the analysis: *ATG32* (involved in autophagy of mitochondria, mitochondria-nucleus signaling pathway and mitochondrial outer membrane, mitochondrion localization [39]), *ATG17* (predicted role in non-selective autophagy [39, 40]), *ATG11* (predicted role in pexophagy [39, 40]), and *ATG8* (putative autophagosome protein; regulates mitochondrial function under ER stress [41]). The expression levels of these genes exhibited wild-type-like patterns in *ksp1*ΔT (Fig. 4G), and in *ksp1*ΔN, we saw potentially elevated transcript levels that did not reach statistical significance. Notably, the background strain *trp*∆/*his*∆/*leu*∆ also showed elevated transcript levels of the genes *ATG32*, *ATG11*, and *ATG8*, albeit only significantly for *ATG32*. This suggests that the disrupted nutrient homeostasis by auxotrophies might also play a role in regulating autophagy gene expression, but the kinase does not significantly affect the transcript level, especially in yeasts that already exhibit some *petite*-like phenotypes. Collectively, these data suggest a role of the Ksp1 kinase in regulating the *petite* formation, but at a population level, the mutant still behaved like *grande* cells in most assays.

### *ksp1*ΔT-induced small colony variants isolated from macrophages are *petites*

**Fig 5:**
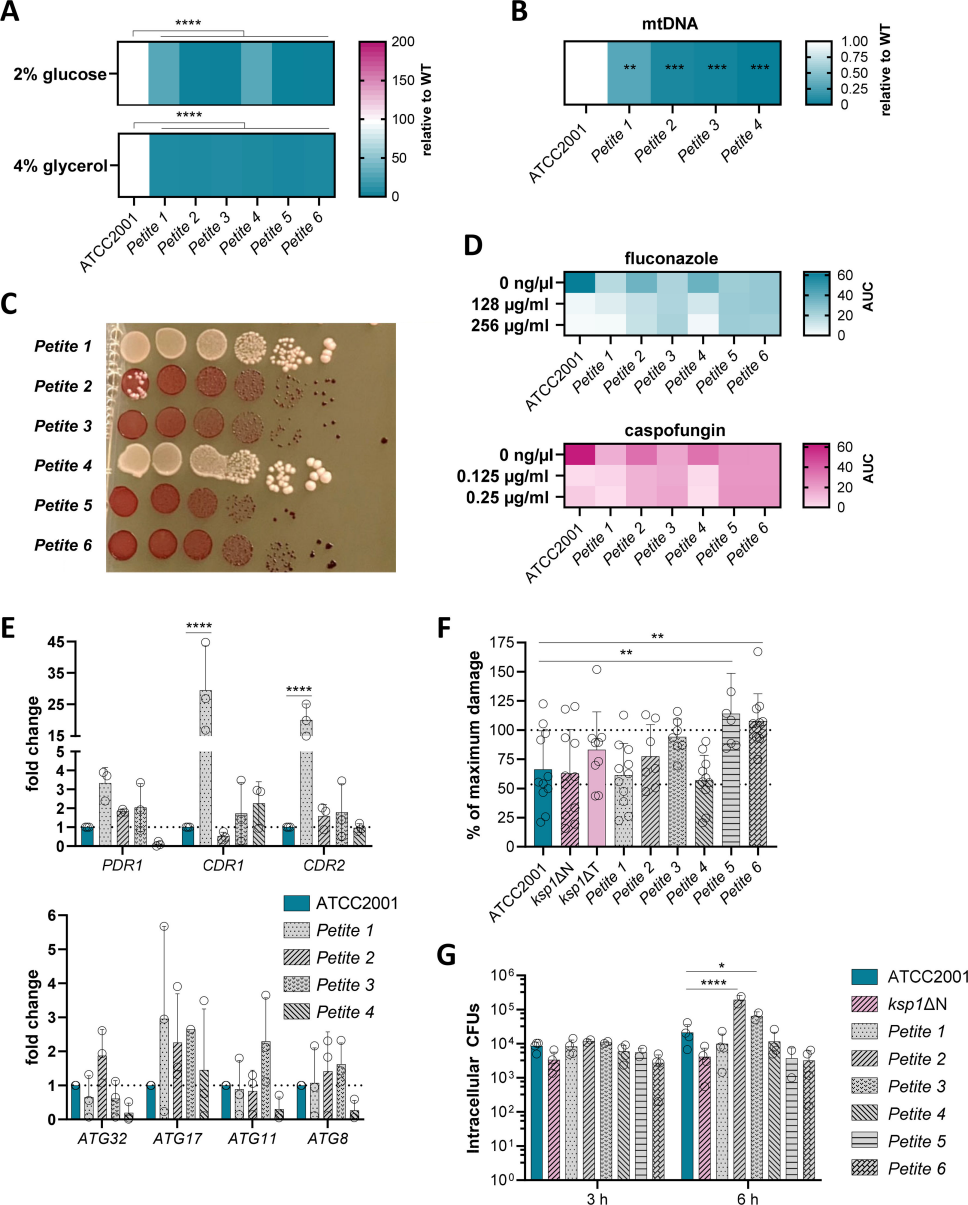
Characterization of the small colony variants isolated from macrophages regarding the *C. glabrata petite* hallmarks. (**A**) Growth of the *C. glabrata* wild type strain and six isolated small colony variants (*petites* 1–6) in SD media supplemented with either 2% glucose or 4% glycerol as the alternative carbon sources at 37°C for 4 days. Growth is shown as the mean area under the curve (*n* = 3). Statistical significance was calculated using a one-way ANOVA with Dunnett’s multiple comparisons test (****, *P* < 0.0001). (**B**) Presence of mitochondrial DNA in the potential *petites* was quantified from overnight cultures via qPCR of the mitochondria-associated gene *COX3* relative to *ACT1* and in comparison to the wild-type ATCC2001 (*n* = 3). Statistical significance was calculated using a one-way ANOVA with Tukey’s multiple comparisons test (**, *P* < 0.01; ***, *P* < 0.001). Asterisks indicate significance compared to the ATCC2001 strain. (**C**) Mitochondrial function was determined by dropping serial dilutions of the potential *petites* on SD+4% glycerol agar containing 0.02% tetrazolium chloride, indicating functional mitochondria by dye reduction. Plates were grown at 37°C for up to 4 days (*n* = 2). (**D**) Antifungal susceptibility of the potential *petites* was measured by performing growth curves in YPD supplemented with the indicated antifungal concentration at 37°C for 3 days. Growth is shown as the mean area under the curve (*n* = 3). (**E**) Expression of efflux pump-related (top) and autophagy-related (bottom) genes of four *petite* strains was determined via qPCR. Gene expression is shown as fold change relative to *ACT1* and compared to ATCC2001. The dashed line indicates no change (*n* = 3). Statistical significance was calculated using a two-way ANOVA with Tukey’s multiple comparisons test (****, *P* < 0.0001). (**F**) Damage induction measured by quantification of lactate dehydrogenase release of the wild-type strain and both *ksp1*Δ kinase mutants, as well as of all potential *petites* to primary human macrophages 24 h post infection (six donors). The lower dashed line indicates the LDH release background measured with an uninfected control. Statistical significance was calculated using a one-way ANOVA with Dunnett’s multiple comparisons test (**, *P* < 0.01). (**G**) Intracellular survival of the *C. glabrata* wild type strain, the *ksp1*ΔN kinase mutant, and all six potential *petites* in primary human macrophages was determined by lysing and then plating intracellular CFUs at the indicated time points (six donors). Statistical significance was calculated using a two-way ANOVA with Tukey’s multiple comparisons test (*, *P* < 0.05; ****, *P* < 0.0001).

As not all *petite* hallmarks were observed for the *ksp1*ΔT and *ksp1*ΔN mutants, we hypothesized that the *C. glabrata ksp1*∆ strains are likely a mix of *petites* and *grande*s, i.e. normal yeasts, which promotes overgrowth by either population under favorable conditions. Thus, we decided to isolate potential *petites* retrieved from macrophages and characterize them further regarding the *petite* key hallmarks (25) (Fig. 5). The *petites* were isolated from YPD plates of *ksp1*∆T (*petites* 2–5) or, as a control for the effect of nutrient stress, *trp*∆ (*petite* 1), which were retrieved either 1 day or 3 days after infecting primary macrophages (see Table 1).

**TABLE 1:** Strains used in this study

Name	Description	Source
ATCC2001	*C. glabrata* reference strain	American Type Culture Collection
*efg1*ΔΔ/*cph1*ΔΔ	*C. albicans* yeast-locked mutant with deletions of the *EFG1* and *CPH1* genes in the SC5314 strain	(29)
*trp*∆/*his*∆/*leu*∆	Deletion of the *HIS3*, *LEU2*, and *TRP1* genes in the ATCC2001 strain	(20)
*trp*∆	Deletion of the *TRP1* gene in the ATCC2001 strain	(20)
*ksp1*∆T	Deletion of the *KSP1* gene using the *TRP1* marker in the *trp*∆ strain, *ksp1*∆::*TRP1 trp1*∆	This study
*ksp1*∆N	Deletion of the *KSP1* gene using the NAT1 resistance marker in the ATCC2001 strain, *ksp1∆::NAT1*	This study
*petite* 1	*trp*∆ retrieved from MDMs 1 day post-infection	This study
*petite* 2	*ksp1*∆ *trp*∆ retrieved from MDMs 1 day post-infection	This study
*petite* 3	*ksp1*∆ *trp*∆ retrieved from MDMs 1 day post-infection	This study
*petite* 4	*ksp1*∆ *trp*∆ retrieved from MDMs 3 days post-infection	This study
*petite* 5	*ksp1*∆ *trp*∆ retrieved from MDMs 1 day post-infection	This study
*petite* 6	*ksp1*∆ *trp*∆ retrieved from MDMs 3 days post-infection	This study
CBS12766	*C. auris* reference strain	Institut Pasteur, Paris
*C. auris ksp1*∆	Deletion of the *KSP1* gene in the CBS12766 strain, *ksp1*∆::*NAT1*	This study

We performed growth curves in 2% glucose as the preferred carbon source, or 4% glycerol as an alternative non-fermentable carbon source, and observed that all isolated potential *petites* showed a significantly reduced growth in both tested carbon sources (Fig. 5A). Quantification of the mitochondrial *COX3* gene revealed that all six potential *petites* had significantly lower levels compared to the wild type (Fig. 5B), indicating a reduced amount of mitochondrial DNA. When performing drop tests with TTC to investigate the mitochondrial reductive power, two of the six potential *petites* grew as white colonies (Fig. 5C), suggesting mitochondrial dysfunction. Importantly, *petite* 2 showed red and white colonies on the TTC agar, indicating a heterogeneous population of mitochondria-deficient and -proficient cells (Fig. 5C). Since this small colony variant was originally isolated as a pure *petite* colony, this could be a sign of a potential reversion to *grande* cells.

Quantification of intracellular ATP levels as a measure of a functional oxidative phosphorylation revealed that two of the six tested *petites* had significantly reduced intracellular ATP concentrations in comparison to the wild type (Fig. 4E). The remaining four *petites* showed large fluctuations in ATP concentrations, potentially due to the lack of a complete correspondence of ATP levels to *petite*/*grande* phenotypes (37) or a spontaneous reversion of the *petites* during some trials. Similarly, MitoTracker staining revealed a range of mitochondrial activity for all tested *petites—*the majority of cells resembled the mitochondria-deficient *mip1*∆ strain (Fig. S5A), while some cells exhibited wild type-like staining. This is most apparent for *petite* 4, which shows a combination of both MitoTracker-positive and -negative cells. As this small colony variant was initially isolated as a purely *petite* colony, it appears that it reverted to *grande* during the experiment. This suggests that the *ksp1*∆T-derived *petite* phenotype is transient and overall less stable than other small colony variants (25, 28).

To analyze whether the *petites* have a decreased susceptibility to echinocandin and azole antifungals, we performed growth curve assays in the presence of different concentrations of either fluconazole or caspofungin (Fig. 5D). As already indicated by the growth with glucose and glycerol, all *petites* grew less than the wild type in medium without antimycotics. In the presence of fluconazole and caspofungin, however, especially *petites* 2, 3, 5, and 6 exhibited augmented growth at all tested concentrations compared to the wild type.

To elucidate the mechanisms underlying the reduced susceptibility observed in these *petites*, we quantified the expression of the efflux pump-related genes *PDR1*, *CDR1*, and *CDR2* via RT-qPCR in *petites* 1, 2, 3, and 4, albeit only *petites* 1 and 4 exhibited mitochondrial dysfunction (Fig. 5C). Notably, *petite* 1 and *petite* 3 demonstrated an increased expression of all three genes relative to ATCC2001 (Fig. 5E), while *petites* 2 and 4 exhibited upregulation of only a subset of genes. Specifically, *petite* 2 showed upregulation of *PDR1* expression as well as slightly elevated transcript levels of *CDR2*, and *petite* 4 demonstrated upregulation of *CDR1* transcription. The somewhat increased gene expression in all of the tested *petites* can explain their decreased antifungal susceptibility. Interestingly, the mitochondrial dysfunction does not seem to correlate with the upregulation of the antifungal resistance genes.

Additionally, RT-qPCR analysis of autophagy-associated genes revealed that all of the tested small colony variants showed a distinct increase in expression for *ATG17* compared to ATCC2001 (Fig. 5E). Additionally, *petite* 2 showed elevated transcript levels of *ATG32*, and *petite* 3 of *ATG11*.

To investigate how these *petites* perform when exposed once more to the hostile environment of the macrophage, we re-infected macrophages with the six *petite* strains. We then analyzed their capacity to lyse the host cells 24 h after infection and assessed their survival within the macrophages at 3 h and 6 h post-infection. Interestingly, *petites* 2, 3, 5, and 6 induced damage similar to the *ksp1*ΔT mutant, which was close to the full lysis control. Notably, *petites* 5 and 6 caused significantly more damage than the wild type (Fig. 5F). Quantification of the intracellular survival in the macrophages revealed that *petites* 2 and 3 showed a significantly increased survival in macrophages at 6 h post-infection (Fig. 5G). The other *petites* did not exceed the wild type level. Thus, some of the *ksp1*ΔT-induced *petites* exhibit an improved virulence in macrophages. Interestingly, the *ksp1*ΔN mutant itself did not cause the same macrophage damage or reach the same intracellular CFU levels as the pre-formed *petites*. This suggests that the formation of *petites*, triggered also by the deletion of *KSP1*, is a prerequisite for these phenotypes, and remaining *grande* cells reduce the observable effect.

In summary, the isolated potential *petites* fulfill the majority of the key hallmarks of that phenotype, indicating that these small colony variants are in fact *petites*. Moreover, this shows that deletion of *KSP1* tips the balance toward *petite* formation and leads to a mixed population of *petites* and *grandes*. Interestingly, the magnitude of the effect seems to depend in part on the deletion method used, by a tryptophan or nourseothricin marker, where stronger effects are often seen with the tryptophan-based protocol. We suggest that this may in part be due to a suboptimal regulation of nutrient homeostasis by a *TRP1* gene put into a different genomic context. In support of this, staining with MitoTracker revealed a reduced fluorescence intensity compared to the wild type for both the *ksp1*∆N mutant and the *trp*∆ mutant. This indicates that not only the deletion of *KSP1*, but also the tryptophan auxotrophy itself can affect the mitochondria (Fig. S5A). In the *ksp1*ΔT mutant, the effect on the mitochondrial functionality is even more pronounced, potentially suggesting an imperfect complementation of the tryptophan auxotrophy. This combination likely leads to the observed phenotype of highly increased *petite* formation rate in this mutant.

### Deletion of *KSP1* can augment the release of pro-inflammatory cytokines in infected macrophages

Based on our data assembled so far, we propose that the observed phenotypes are associated with an altered immune response of host macrophages. Therefore, we monitored the production of defined cytokines by the macrophages during infection with the *ksp1*ΔT mutant (Fig. S6). After 24 h, *ksp1*∆T induced significantly more IL-1β release than the wild type (Fig. S6A), reflecting the strong phagocyte lysis caused by this mutant (Fig. 3B). While the *ksp1*∆N strain did not trigger IL-1β release above wild type level, both *ksp1*∆ mutants led to an augmented release of the pro-inflammatory TNFα, although only the increase due to *ksp1*∆T was statistically significant (Fig. S6B). This trend is inverted for the pro-inflammatory IL-8, with both *ksp1*∆ mutants exhibiting a lower release compared to the wild type (Fig. S6C). This discrepancy was only statistically significant for the *ksp1*∆N mutant. IL-6 release, in contrast, was not affected upon *ksp1*∆T infection, although the *ksp1*∆N mutant induced a more modest macrophage reaction (Fig. S6D). Notably, the *ksp1*∆T infection induced a significantly stronger release of GM-CSF than the wild type (Fig. S6E), a cytokine, which leads to the recruitment of monocytes to the site of infection and activates macrophages (21). The *ksp1*∆N mutant only elicited GM-CSF production at wild type levels.

Overall, the deletion of *KSP1* seems to induce a stronger cytokine release during the *C. glabrata*-macrophage interaction, especially in the tryptophan marker-based deletion strain, which seems more prone to form *petites*. This is not solely due to a potentially incomplete reversion to tryptophan auxotrophy, as the *trp*∆ control strain did not exhibit this phenotype. Again, it seems that the effect of *KSP1* deletion may be augmented by subtle differences in the nutrient homeostasis.

### Role of Ksp1 in *C. glabrata*’s survival in human blood and in the *C. auris*-macrophage interaction

**Fig 6:**
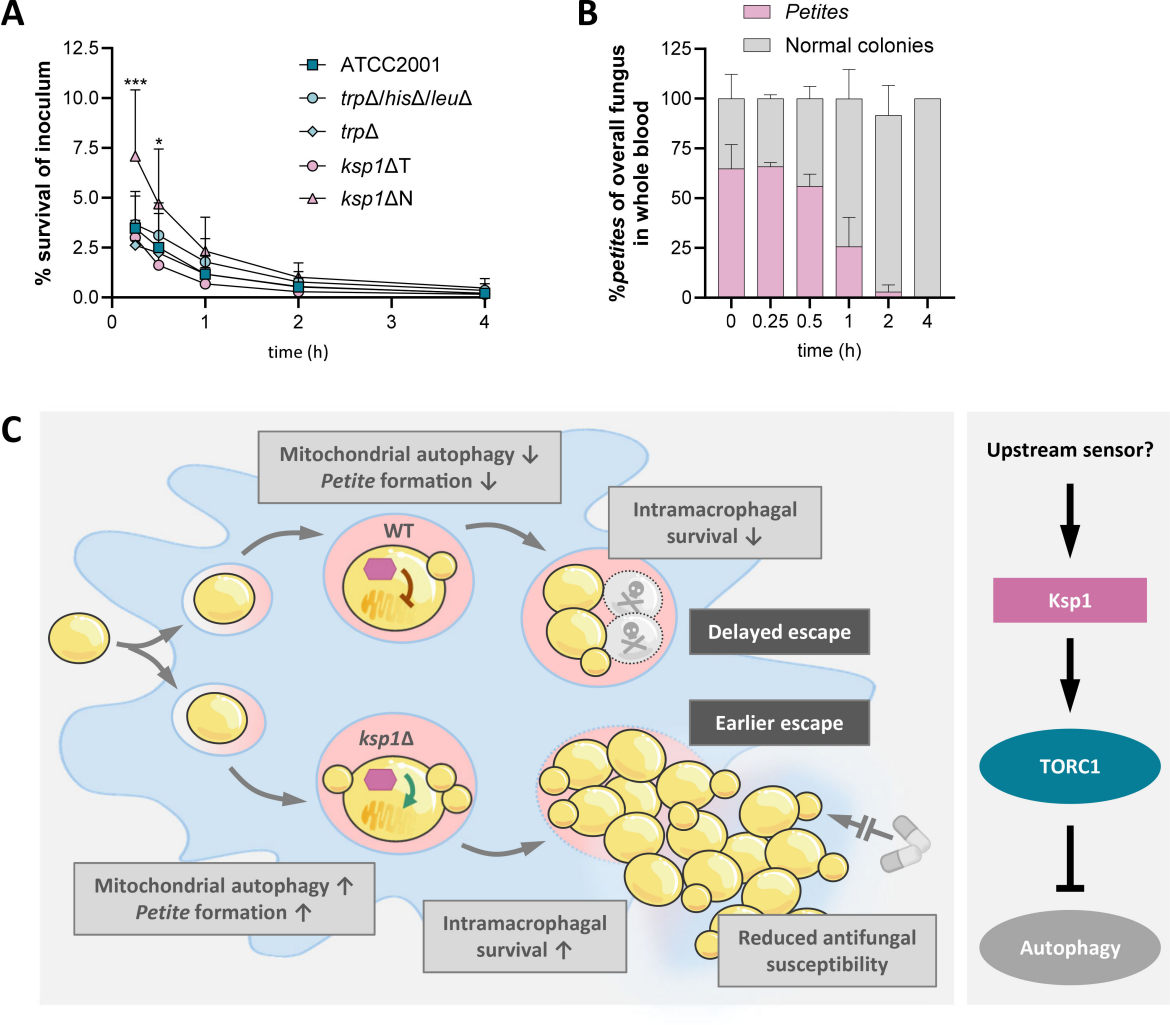
Survival of the *ksp1*Δ kinase mutant in *ex vivo* whole blood and how Ksp1 regulates the intramacrophage *petite* formation. (**A**) Survival of the three parental *C. glabrata* strains, the *ksp1*∆T kinase mutant from the kinase library, and the independently constructed *ksp1*∆N mutant in whole human blood was assessed over the course of 4 h by plating surviving CFUs. Survival is shown relative to the inoculum (*n* = 4 donors). Statistical significance was calculated using a two-way ANOVA with Dunnett’s multiple comparison test (*, *P* < 0.05; ***, *P* < 0.001). Asterisks indicate significance compared to the ATCC2001 strain. (**B**) Percentage of *petites* in relation to the overall counted CFUs of the *ksp1*∆T mutant at each time point plated during the whole blood experiment (*n* = 4 donors). (**C**) (Left panel) Model of how Ksp1 regulates the *C. glabrata*-macrophage interaction. With *KSP1* being present, it negatively regulates mitophagy, leading to more mitochondria-proficient *C. glabrata* cells in the macrophage. Hence, the ratio of normal yeast cells vs*. petites* tips toward the less adapted *grande* cells, decreasing the overall intramacrophagal survival. As the intracellular fungal load affects the time point of escape, this leads to a delayed escape. In case of a deletion or physiological downregulation of *KSP1*, mitophagy is not negatively regulated, leading to more cells with mitochondrial dysfunction, which then become *petite*. The ratio of overall CFUs inside the macrophage tips toward more *petite* cells with increased survival inside the phagocyte. When these *petites* revert and start replicating after this persistence phase, this leads to an earlier escape due to the higher intracellular fungal mass. Within this escaping mixed population are *petites* with reduced antifungal susceptibilities. (Right panel) Regulatory circuit known from *S. cerevisiae* (35). We propose that a similar regulatory circuit is happening in *C. glabrata* with an unknown trigger, which could activate the Ksp1 kinase, leading to a downregulation of mitophagy. In case the trigger is missing or represses the Ksp1 function, mitophagy can take place.

*C. glabrata petites* have previously been isolated from candidemia patients (36). Considering the observed role of Ksp1 to regulate *petite* formation, we hypothesized that the Ksp1 kinase might also play a role in *C. glabrata*’s survival in human blood. Hence, we infected whole human blood *ex vivo* with the isogenic wild type strains and both *ksp1*∆ mutants and then quantified their survival. Remarkably, only the nourseothricin marker-based *ksp1*∆N mutant exhibited a significantly higher level of survival in whole blood compared to the ATCC2001 strain (Fig. 6A). The *ksp1*∆T strain and the auxotrophic control strains demonstrated a survival pattern similar to that of the wild type, and the *ksp1*∆T mutant was the only strain with visible *petites* within the surviving colony-forming units. We calculated the proportion of *petites* among the total recovered *ksp1*∆T yeast cells. Notably, the number of *petites* declined gradually over the course of the whole blood infection (Fig. 6B), which suggests that the *petite* phenotype is less conducive to the survival of *C. glabrata* in whole blood and gets outcompeted by *grande* cells, which could compensate any benefit the strain gained from the *KSP1* deletion that increased survival of the *ksp1*∆N strain. In support of this, the isolated *petites* themselves showed survival either similar to or lower than the ATCC2001 strain in whole blood (Fig. S7A). Collectively, our results indicate that Ksp1-induced *petites* are not essential for the survival of *C. glabrata* in human blood, and that the role of this kinase is rather restricted to macrophage environments and potentially other yet untested niches (Fig. 6C).

As the Ksp1 kinase seems to be a key regulator for a delayed escape of *C. glabrata* cells, dependent on the genomic context of *TRP1*, we finally investigated the function of gene analogs in another emerging pathogenic *Candida* species, *Candida auris* (*Candidozyma auris*). *C. auris* has been shown to be phagocytosed by macrophages and to successfully escape after 8–10 h (42). Deletion of *KSP1*, however, did neither affect *C. auris* damage induction (Fig. S7B) nor survival and replication inside macrophages (Fig. S7C), indicating that the role of the Ksp1 kinase is not universal among pathogenic yeasts.

In summary, our study identified Ksp1 as a previously unrecognized regulator of delayed macrophage escape in *C. glabrata*. In our model of events (Fig. 6C), we suggest that it regulates, controlled by an unknown sensor and (in our experiment) likely variations in tryptophan levels, the level of autophagy of phagosome-trapped yeasts. When its activity is lowered, increased mitophagy leads to reversible loss of mitochondria and thereby the formation of more antifungal- and phagosome-resistant *petites*. Through this regulation of autophagy, Ksp1 also controls the kinetics of the final fungal exit from the engulfing macrophages.

## DISCUSSION

Several studies have proposed that *C. glabrata* may exploit macrophages as Trojan horses to hide from more effective immune activities (12, 22, 23, 43). Initial evidence showed a prolonged intracellular persistence of *C. glabrata* cells inside these phagocytes for up to 7 days (24, 25). However, the mechanisms that enable *C. glabrata* to establish such persistence, as well as the specific fungal processes involved, have not yet been fully elucidated, but some relevant observations have been made: On the fungal side, the oxidative stress response has been linked to a reduced long-term survival in macrophages (44). On the host side, some evidence suggests that the macrophage Syk kinase—an enzyme involved in phagosome maturation—plays a role in the persistence (45). Notably, after deletion of *SYK*, *C. glabrata*’s intracellular replication is triggered within 4 h, whereas in wild type macrophages, the fungus remains undivided for up to 18 h (46). Until now, it has remained unclear whether *C. glabrata* is actively inducing its intracellular persistence or whether the host directs the fungus into this state, delaying its eventual escape from the macrophage.

With this study, we sought to answer the question of how the delay in escape of *C. glabrata* is regulated. Since the dimorphic fungus *C. albicans* does not persist in macrophages as long and instead escapes within a few hours via hypha formation, we hypothesized that the comparatively longer intracellular persistence of the yeast-only *C. glabrata* is due to its differences in morphology. Previous findings seem to support this notion: yeast-locked *C. albicans* mutants survive inside macrophages in a zebrafish model for 40 h and disseminate intracellularly to other organs (47). However, using human primary macrophages at physiological temperature, we found that a yeast-locked *efg1*ΔΔ/*cph1*ΔΔ *C. albicans* mutant exits no later than 24 h after infection, following a robust intracellular replication phase. In contrast, *C. glabrata* escaped these primary phagocytes only after 2–3 days, indicating that the delay in escape is not due to replication in the yeast form *per se*. Our findings for *efg1*ΔΔ/*cph1*ΔΔ are in good agreement with a previous study, which described for it a pronounced replication phase between 4 and 12 h, resulting in an enlarged phagosome (17). However, the same study also found similar exit dynamics for *C. glabrata* (17), which contradicts our observed delay in escape. This discrepancy may be due to different macrophage models, as the prior study used a less potent murine macrophage-like cell line, whereas we employed primary human macrophages.

Following a robust intracellular replication phase, fungi that grow in a yeast morphology within host cells—including *Cryptococcus neoformans*, *Candida auris*, and *Histoplasma capsulatum*—can escape from macrophages through various mechanisms (13, 22). *C. auris* induces metabolic stress, ultimately killing the macrophage (42), whereas *C. neoformans* and *H. capsulatum* trigger apoptosis to enable their exit (48–50). *C. neoformans* can additionally escape macrophages via non-lytic exocytosis (51–53). Both *C. neoformans* and *H. capsulatum* can also escape via mechanical membrane rupture once a substantial intracellular fungal burden is reached (50, 54). A similar mechanism has been proposed for *C. glabrata*, as macrophages with a high fungal load eventually burst (our data and reference 15). In support of this mechanistic proposition, we have established a direct link between fungal burden and macrophage rupture, showing that higher intracellular *C. glabrata* cell numbers accelerate escape.

Having ruled out that the inability to produce hyphae is the main reason for the delayed exit of *C. glabrata*, we investigated whether the macrophages dictate the delayed exit trajectory of the fungus. Interestingly, the transcriptional responses of macrophages following infection with either *C. glabrata* or the yeast-locked *efg1*ΔΔ/*cph1*ΔΔ *C. albicans* mutant remained strikingly similar up to 32 h, suggesting that the macrophages do not contribute to slowing the exit event. Such a uniform early host response has also been seen for other filamentous and non-filamentous *Candida* species of the CUG clade (55). Notably, the phagocyte response even seemed to be independent of fungal viability in these cases and was likely driven by the recognition of conserved cell wall components (55). While the overall composition of the cell wall is similar in *C. albicans* and *C. glabrata*, its protein content, spatial organization, and exposure differ significantly (56, 57). This suggests that the remarkably similar macrophage response observed in our data is likely attributable to an unspecific recognition of common cell wall components or the yeast shape itself, shared by both fungi.

Differences in the macrophage response were observed at later time points, mainly in the form of the hypoxia response, which was much stronger with the *efg1*ΔΔ/*cph1*ΔΔ mutant than with *C. glabrata*. A likely explanation for these differences is a stronger activation of the macrophages at these later time points, specifically with the *C. albicans* mutant, leading to a strong Warburg effect (58). An interesting alternative hypothesis offers itself in the form of differences in the mitochondrial activity of the phagocytosed yeasts. If *C. glabrata* takes a path of reduced mitochondrial activity (as described below), this could lead to lower overall oxygen consumption and alleviate a hypoxic response that could be triggered by the metabolic activity of the phagocytosed fungus.

Our findings up to this point indicate that the delayed escape of *C. glabrata* cells is fungal-driven. Our fungal mutant library screen in macrophages identified several kinases, among them especially Ksp1, the deletion of which accelerated macrophage cell lysis and led to a significantly increased fungal survival. Moreover, the *ksp1*∆T strain readily formed small colony variants, or *petites*, inside macrophages at much higher frequencies than previously observed for the *C. glabrata* wild type (25). *Petites* are characterized by mitochondrial deficiency, increased antifungal and stress resistance, and, importantly in this context, augmented survival in phagocytes (25). In *C. glabrata*, we were able to show that *KSP1* deletion in a strain with intact nutrient homeostasis results in upregulation of the mitophagy gene *ATG32*, suggesting that *petite* formation is driven by a normally Ksp1-suppressed increase in fungal mitophagy. Moreover, the *ksp1*∆T strain contained smaller mitochondria, contributing to the reduced mitochondrial functionality.

A link between the Ksp1 kinase and autophagy regulation was first identified in the baker’s yeast *S. cerevisiae* (35). Here, Ksp1 suppresses autophagy by activating the nutrient-signaling Tor kinase complex 1 (TORC1), a process partially mediated by the Ras/cAMP-dependent protein kinase A pathway (35). In nutrient-rich conditions, Ksp1-activated TORC1 inhibits autophagy. Under stress, however, TORC1 is inactivated, allowing autophagy to proceed (59, 60). We propose that in *C. glabrata*, Ksp1 is regulated by an upstream sensor that monitors the yeast’s environment (Fig. 6C). Before macrophage internalization, *C. glabrata*’s environment is comparatively rich in nutrients, which would trigger activation of Ksp1 and subsequently TORC1. This cascade inhibits mitophagy, preventing the formation of *petites* and ultimately reducing *C. glabrata*’s survival under the upcoming starvation and stress conditions of the maturing phagosome (61) (Fig. 6C). Wild-type *C. glabrata* cells may sense this shift inside the phagosome, leading to Ksp1 and TORC1 inactivation and a subsequent increase in mitophagy. The transient decline in mitochondrial functionality, due to their reduced numbers, would promote *petite* formation, which enhances intracellular survival of *C. glabrata* (Fig. 6C). Later, when the immediate starvation has been overcome, and Ksp1 is active again, the yeast cells could undergo mitochondrial replication. After they regain full mitochondrial proficiency, they rapidly proliferate, generating a substantial fungal burden and facilitating macrophage lysis.

Supporting our findings on Ksp1-regulated mitochondrial autophagy and its impact on *C. glabrata*’s survival and escape from macrophages, previous studies have demonstrated that autophagy of *C. glabrata* cells is induced upon phagocytosis, and that this promotes intracellular survival (33, 40). Moreover, transcriptional analyses comparing *petites* and non-*petites* inside of THP-1-derived macrophages have revealed an upregulation of autophagy processes, specifically mitochondrial autophagy, in the small colony variants (28).

We observed a specific temporal pattern in the presence of *ksp1*∆T *petites* within the phagosome, from an initial peak at 3 h, to undetectable levels at 6 h, and back to high frequency at 24 h. A possible explanation is that the high mitophagy upon first contact with the harsh phagosomal environment—unmodulated due to lack of Ksp1—triggered an initial high rate of *petite* formation. While these *C. glabrata petites* still had available nutrient reserves, they were outcompeted by the remaining *grandes*, only to become the better adapted form again at later time points when nutrients became even more limiting. This time point may also represent a critical stage in the pathogen’s intracellular lifecycle, potentially marking a decision point between the prolonged macrophage persistence and initiation of escape. A detailed dissection of these events may, in the future, give more insight into the triggers and signaling events that guide this decision.

A large number of yeast species possess the ability to form slow-growing *petite* cells by spontaneous loss of respiratory functions (62, 63). For *C. glabrata*, prior research has shown that mutations in genes of mitochondria-associated pathways (28, 36), as well as loss of mitochondrial DNA, can induce the small colony variant state (25). Here, we have identified an additional mechanism mediated by mitophagy, which may represent a more transient way of forming *petites*, as the number of mitochondria can quickly be recovered through replication and division of the organelle (64, 65). This transient switch between the *petite* and non-*petite* state would allow *C. glabrata* to finely regulate its escape from macrophages—either adopting a delayed exit trajectory that favors a prolonged intracellular persistence or facilitating a more rapid escape when necessary. The environmental cues that lead to either decision remain unclear and warrant further investigation but are likely linked to a changing nutritional state or oxidative stress experienced by yeast cells. We already found hints toward nutrient starvation influencing the *ksp1*∆-derived *petite* phenotype, as we show that even a mutant where the *TRP1* gene was used as a deletion marker, resulting in an altered genomic context, showed an increased mitochondrial deficiency. We suggest that this is due to subtle misregulation of the tryptophan biosynthesis in this mutant, which, maybe serendipitously, enhanced the nutrient-dependent *ksp1*∆ phenotypes even further in one of our two mutants.

Either way, the induction of the *petite* phenotype in *C. glabrata* appears to affect predominantly the fungal side of the interaction, as the macrophage transcriptomic responses to *petites* and non-*petites* have earlier been shown to be largely indistinguishable (28). While this was found with *petites* induced by mitochondrial mutations, it is likely also true for Ksp1-induced *petites*.

We observed the Ksp1-regulated mitophagy in the tryptophan marker-based mutant. As described above, this may have subtly exacerbated the nutrient stress of *C. glabrata* in the phagosome and thereby further increased the Ksp1 deletion-induced *petite* formation, as shown with the MitoTracker staining. The precise role of this and other amino acids in intraphagosomal *petite* formation and survival remains to be elucidated. Nevertheless, it has been shown that the slow growth of *S. cerevisiae petites* is largely due to perturbations in their amino acid metabolism (37). Additionally, bacterial small colony variants are often auxotrophic for aromatic amino acids, including tryptophan (66), suggesting a link between the *petite* phenotype and tryptophan.

Macrophages infected with the *ksp1*∆T mutant also exhibited a more pronounced induction of proinflammatory cytokines—specifically IL-1β and TNF-α—compared to those infected with the wild type, suggesting that the Ksp1 kinase of *C. glabrata* may attenuate immune activation. Moreover, this stronger cytokine response may be attributable to the high proportion of *petites* present, especially in this mutant, as these variants have been shown to trigger a pro-inflammatory transcriptional state in THP-1 macrophages (28). Furthermore, infection with the *ksp1*∆T mutant led to an increased release of GM-CSF. A strong GM-CSF release (compared to other yeasts such as *C. albicans* or *S. cerevisiae*) has been observed previously for *C. glabrata* (15). Given that the *ksp1*∆T mutant produces a high proportion of *petites*, it is intriguing to speculate that the *petite* phenotype further augments GM-CSF production, thereby recruiting more macrophages to the site for *C. glabrata* to infect. Alternatively, the Ksp1 kinase may normally function to temper the GMCSF release, ensuring that only a limited number of macrophages are attracted—sufficient to provide space for intracellular survival without provoking an overwhelming immune response that could lead to fungal clearance.

The survival advantage conferred by Ksp1-induced *petites* appears to be specific to the macrophage phagosomal niche. In whole blood infected with the *ksp1*∆T mutant, the number of recovered *petites* declined, indicating that this phenotype may not be beneficial in that specific environment. Although *petites* have been isolated from candidemia patients (25, 28, 36), *in vivo* studies in mice consistently reveal a lower fungal burden for these small colony variants (28, 36), implying that the *grande* state is generally more favorable for *in vivo* survival. This is further supported by observations that no *petites* emerge when sterile blood cultures are spiked with *C. glabrata* (36). Notably, *petites* isolated from candidemia patients were frequently recovered following treatment with azoles or echinocandins (28, 36), and studies in mice suggest that under echinocandin treatment, the *petite* phenotype may confer a survival advantage (28). In alignment with these findings, *C. glabrata*’s small colony variants have been shown not to be affected by echinocandins (28). Moreover, *petites* demonstrate decreased susceptibility to azoles (25, 28), and the *petite* phenotype can even be induced by fluconazole treatment, conferring a cross-protection against phagocytes (25). Similarly, both the *ksp1*∆ mutant itself and the Ksp1-induced *petite* phenotype led to decreased susceptibility to fluconazole and caspofungin, suggesting a broader Ksp1-derived survival strategy of *C. glabrata*. The precise mechanisms underlying the resistance observed in the *ksp1*∆ mutant and *petites* remain to be elucidated—and whether a reduced number of mitochondria in an otherwise *grande ksp1*∆ mutant strain may already suffice to trigger these effects. Previous studies have identified an upregulation of the efflux pump-related genes *PDR1* and *CDR1*, providing protection from fluconazole in *petites* (25, 28), which we confirmed in some of the *ksp1*∆T-induced small colony variants.

Our analyses did not reveal all hallmarks of the *petite* phenotype in the *ksp1*∆T-derived small colony variants. As noted above, it is also conceivable that only a subset of the *C. glabrata* population transitions to the *petite* state, given that mitophagy only exerts a transient effect on mitochondrial function. Consequently, in a form of bet-hedging strategy, a heterogeneous population of *petite* and *grande* cells may arise, with each subpopulation potentially prevailing under different environmental conditions. While the *grande* subpopulation proliferates better in the presence of non-fermentable carbon sources or survives better within whole blood, the *petite* subpopulation has survival and growth advantages in the presence of macrophages. Thereby, the relative distribution of these subpopulations may change with the fluctuating environments throughout the infection process. Such mixed populations have been documented previously in *C. glabrata* (28) as well as in bacterial small colony variants (67), which complicates the identification of *petites* in clinical and laboratory settings.

Small colony variants are frequently seen in bacteria such as *Staphylococcus aureus* (68), where they can be recovered intracellularly and from patient samples (69). In contrast, among yeasts, such variants have been reported—to our knowledge—so far only in *C. glabrata* (25, 28), *S. cerevisiae* (25, 37, 70), and *C. albicans* (71). Given that *C. auris* is phagocytosed by macrophages and escapes at a slower pace compared to *C. albicans* (42), we investigated whether this emerging pathogen might employ a similar Ksp1-driven strategy. However, deletion of the *KSP1* homolog did not affect the *C. auris*-macrophage interaction. This outcome is not entirely surprising, as *C. auris* has a markedly different approach for intracellular replication and survival. In this regard, *C. auris* more resembles *C. albicans* and escapes from macrophages within a few hours (42) rather than days, as for *C. glabrata*. Moreover, the putative Ksp1 kinase protein sequence in *C. auris* shares only 34% similarity with *C. glabrata*’s ortholog (candidagenome.org), indicating potentially different roles in both species. Additionally, a previous study has suggested that *C. auris* may be a *petite*-negative yeast that does not tolerate loss of mitochondrial DNA or respiratory function (72). Collectively, these observations indicate that the Ksp1-regulated delay in macrophage escape—and its effect on antifungal resistance—is a specific mechanism that *C. glabrata* employs as part of its overall strategy as a successful human pathogen.

## MATERIALS AND METHODS

### Fungal strains and culture conditions

The strains used in this study are depicted in Table 1.

For all experiments, single colonies were picked from yeast extract peptone dextrose (YPD) agar plates and grown overnight in liquid YPD medium in an orbital shaker at 180 rpm at 37°C for *C. glabrata* and 30°C for *C. albicans* and *C. auris*. Yeast cells were then harvested by centrifugation (20,000 × *g*, 1 min), washed twice with phosphate-buffered saline (PBS), and the yeast cell number was adjusted to the value required in the experiment.

### Generation of deletion mutants

All primer sequences were designed manually using NCBI Primer-BLAST (73) and Serial Cloner. Primers were designed and named using the same nomenclature and rules as in reference 74. Barcode tags were added to primers based on those used for knockout of the orthologous genes in *Saccharomyces cerevisiae* (75). Oligonucleotides were either commercially purchased in 96-well plate format (ThermoFisher, France) or synthesized in-house using DNAscript SYNTAX platform (France).

The *TRP1* gene was used as a selection marker for the construction of kinase knock-out mutants in the *C. glabrata* ATCC2001 *trp1*∆ strain (20). We used fusion PCR to generate the deletion cassettes. In a first step, 500 bp DNA fragments located upstream or downstream of the target ORF were amplified from *C. glabrata* genomic DNA using primers harboring, at their 5′ end, constant U1 and D1 sequences (Table S1), while *TRP1* was amplified from plasmid pCgACT-P_TDH3_-GTW (74) using tripartite primers with (i) constant U1 or D1 sequences, (ii) gene-specific barcode tags, and (iii) 5M or 3M sequences hybridizing to the *TRP1* gene. The conditions for a 50 µL reaction were as follows: 1× Taq buffer, 0.2 µM dNTPs, 0.5 µM each primer, 1 µL Taq-Polymerase; 94°C for 10 min, 50 cycles 94°C for 30 s, 55°C for 30 s, 68°C for 30 s for flanking DNA fragments and 2 min for *TRP1*, finally 10 min at 72°C. Fusion PCR was then carried in a 4 × 100 µL volume with the same conditions as above: 1× long range DNTPack buffer, 0.2 µM dNTPs, 0.5 µM each primer, 0.5 µL long-range DNTPack (Roche) and 1 µL PCR containing flanking fragments, 4 µL of PCR containing *TRP1*; 94°C for 10 min, 5 min of elongation at 68°C followed by 35 cycles 94°C for 30 s, 55°C for 30 s, 68°C for 3 min, finally 10 min at 68°C. The final deletion construct was purified by ethanol precipitation. Alternatively, we used the *NAT1* gene amplified from plasmid pV1093 (76) as a selection marker. The same protocol was applied except for the use of oligonucleotides devoid of barcodes and hybridizing to *NAT1* instead of *TRP1*.

*C. glabrata* ATCC2001 *trp1*∆ was used to establish the collection of kinase knock-out mutants. From overnight cultures, subcultures in 50 mL YPD were inoculated at an OD_600_ of 0.1 and grown for 5–6 h at 30°C to reach an OD_600_ of 0.4–0.8. Hereafter, yeast cells were kept at 4°C. Yeast cells were washed twice in 2 mL TE (Tris 0.1 M, pH 7.5; EDTA 10 mM, pH 8) and once in 1 mL LiAc/TE (1× TE, pH 7.5; LiAc 0.1 M, pH 7.5). Cells were resuspended in 200 µL LiAc/TE. 50 µL of *C. glabrata* cells were then incubated for 30 min at 30°C in 300 µL 1× PEG (1× TE, pH 7.5; LiAc 0.1 M, and 40% PEG 4000) containing 5 µL of salmon sperm DNA (10 mg/mL) and 10 µL of the fusion PCR fragment. Heat shock was performed in a water bath at 42°C for 15 min, cells were centrifuged at 4,000 rpm for 5 min, and resuspended in 100 µL water. Finally, yeast cells were plated on synthetic complete (SC) agar lacking tryptophan and grown at 37°C for 2 days.

*C. auris* strain 5175 was transformed using a slightly different protocol; specifically, heat shocks were performed in a water bath at 44°C for 15 min, cells were centrifuged at 4,000 rpm for 5 min, and re-suspended in 100 µL water. Finally, yeasts were plated on SC containing 200 µg/mL nourseothricin and grown at 37°C for 2 days.

To construct the independent *ksp1*∆N mutant, the open reading frame (CAGL0F03311g) was replaced with a NAT1 resistance cassette in the strain ATCC2001.

### Growth assays to assess mitochondrial activity

Similar to a previous publication (25), *C. glabrata* cells were adjusted in a 96-well plate to 1 × 10^4^ yeast cells per well in 200 µL SD medium (1% yeast nitrogen base, 0.5% ammonium sulfate) either supplemented with 2% glucose or, as a non-fermentable carbon source, 4% glycerol. Growth was assessed as absorbance at 600 nm at 37°C for 4 days in a multiwell plate reader (Infinite M200 PRO plate reader; Tecan Group GmbH) with orbital shaking.

Mitochondrial activity was tested by dropping 5 µL of serially diluted yeast cultures on solid YPD agar supplemented with 0.02% TTC (2,3,5-triphenyltetrazolium chloride) (Sigma-Aldrich). As differences in fermentation capability can affect the TTC test, further drop tests were performed on SD agar plates (1% yeast nitrogen base, 0.5% ammonium sulfate, and 2% agar) either supplemented with 2% glucose or 4% glycerol. Pictures of the drop tests were taken at the indicated time points after incubation at 37°C.

### Antifungal susceptibility test

*C. glabrata* cells were adjusted in a 96-well plate to 1 × 10^4^ yeast cells per well in 200 µL YPD supplemented with increasing fluconazole (4, 8, 16, 32, 64, 128, and 256 mg/mL) or caspofungin (0.125, 0.25 µg/mL) concentrations. Susceptibility was tested by absorbance measurement at 600 nm at 37°C continuously for 75 h.

### Culture of J774A.1 macrophage-like cells

The J774A.1 macrophage-like cells (mouse BALB/c monocyte macrophage, ATCC, #ATCC-TIB-67) were routinely cultivated at 37°C and 5% CO_2_ in RPMI 1640 (Gibco, Thermo Fisher Scientific) supplemented with 10% fetal bovine serum (FBS) (Bio&Sell) for no longer than 15 passages. For propidium iodide screenings, J774A.1 cells were seeded at a total concentration of 4 × 10^4^ cells/well in a 96-well plate and incubated overnight at 37°C and 5% CO_2_. Prior to the infection, the old medium was aspirated, the macrophages were washed once with pre-warmed PBS, and RPMI without FBS was added.

### Propidium iodide screening

Live-cell imaging of the host cell lysis dynamics was performed as described previously (77). Briefly, *C. glabrata* strains were prepared as described above, and J774A.1 cells were infected with an MOI of 5. 4 µg/mL propidium iodide was added to each well. The infection was imaged in a Zeiss Celldiscoverer 7 for 24 h at 37°C and 5% CO_2_. Pictures were taken every 30 min at 5 × 2 magnification from four different positions per well in bright field, and fluorescence was measured in the DsRed filter (wavelength 555 nm). Microscopy pictures from the red channel were exported and analyzed using Fiji (78). Using the threshold function, images were converted to binary images, and the number of PI-positive nuclei was counted for each image using macro batch analysis and the Particle Analyzer tool. The counts of the PI-positive J774A.1 cells for each *C. glabrata* mutant were calculated as a percentage of the PI counts for the *C. glabrata* wild-type strain ATCC2001.

### Monocyte isolation from buffy coats and macrophage differentiation

Human peripheral blood was collected from healthy volunteers. Preparation of human monocyte-derived macrophages (hMDMs) was performed as described before (79) based on the selection of monocytes by magnetic automated cell sorting of CD14-positive monocytes and a differentiation period of seven days. Adherent hMDMs were detached with 50 mM EDTA in PBS and seeded in 96-well plates (4 × 10^4^ hMDMs/well) in RPMI supplemented with 10% FBS and 50 ng/mL M-CSF (ImmunoTools) and incubated overnight. Incubation of hMDMs was always performed at 37°C and 5% CO_2_. Before infection with *C. albicans*, the previous medium was removed, hMDMs were washed once with pre-warmed RPMI, and RPMI without FBS was added.

### Primary macrophage infections to assess damage induction, intracellular survival, and replication

*C. glabrata* strains were prepared as described above, and hMDMs were infected with an MOI of 5 or an MOI of 1 (indicated in the figure legends). To assess damage induction, after 24 h of infection, the release of the cytoplasmic enzyme lactate dehydrogenase (LDH) was measured as a marker for necrotic epithelial damage (3) using a Cytotoxicity Detection Kit (Roche) according to the manufacturer’s protocol. The LDH release was calculated as a percent of a full lysis control, where maximum LDH release was induced by the addition of 0.5% Triton X-100 to uninfected macrophages for 10 min.

To determine intracellular fungal survival and replication 3 h and 6 h post infection, the supernatant was removed and macrophages were lysed by adding 200 µL ddH_2_O, scraping the well, and pipetting up and down to break the cells. This was repeated with each well five times. The supernatant and lysate were each appropriately diluted with PBS and plated on YPD plates. The plates were incubated at 37°C, and CFUs (overall colony numbers and *petite* colonies) were determined after 1 day and 4 days to assess fungal survival and intracellular replication.

To study the escape of *C. glabrata*, a previously published persistence model (25) was adjusted. Briefly, hMDMs were either seeded in a 24-well plate (2 × 10^5^ hMDMs/well) for fungal CFU plating or in a six-well plate (1 × 10^6^ hMDMs/well) to take samples for RNA isolation and additionally plate the fungal CFUs. Importantly, the macrophages were not infected in RPMI without FBS, but in RPMI supplemented with 10% human serum (Bio&Sell, human serum, AB male, sterile-filtered, lot. BS.321496.5D). *C. glabrata* was prepared as described above, and the macrophages were infected with an MOI of 1. After 3 h, the medium (pre-warmed RPMI + 10% HS) was exchanged in all wells except for those for the 3 h time point (1 mL for 24-well plates, 3 mL for 6-well plates). Afterward, the medium was exchanged daily: for the 24-well plate, 500 µL was replaced with fresh RPMI + 10% HS. For the six-well plate, 1 mL was exchanged. To plate the CFUs, the supernatant was collected in a 50 mL conical tube. The remaining extracellular yeast was retrieved by one wash step with PBS and combined with the supernatant. To collect the intracellular CFUs, the macrophages were lysed by adding 0.5% Triton X100 for 10 min. After three subsequent wash steps with water, which were all combined in a 50 ml conical tube, the lysate and supernatant were appropriately diluted with PBS and plated on YPD plates. The plates were incubated at 37°C, and CFUs were counted after 1 day.

In general, after counting the CFUs after 1 day, all YPD plates were incubated for a further 3 days at 37°C to check for *petites*. The *petite* frequency was determined similarly to a previous publication (25), but in a 96-well plate (MOI 1). For that, the same survival and replication assay was performed, but instead of plating only after 3 h and 6 h, 24 h and 72 h time points were added for plating the intracellular fungal cells. All YPD plates were incubated for at least 4 days at 37°C. Appearing small colonies were re-streaked on YPD agar for further characterization.

### DNA isolation and check for the presence of mitochondrial DNA

DNA was isolated from the *C. glabrata* strains, which were grown in YPD overnight at 37°C and 180 rpm. The cultures were collected in 50 mL conical tubes and centrifuged for 5 min at 4,000 rpm. The pellet was resuspended in distilled water, and cells were centrifuged again at 13,000 rpm for 2 min. The pellet was resuspended in proteinase buffer (10 mM Tris-Cl, pH 7.5; 50 mM EDTA, pH 7.5; 0.5% SDS; 1 mg/mL proteinase K). Cells were incubated for 30 min at 60°C. 0.8 mL phenol/chloroform/isoamyl alcohol was added, the samples were vortexed for 4 min, and centrifuged at 13,000 rpm for 3 min. To precipitate the gDNA, the hydrophilic phase was added 1:1 to ice-cold isopropanol and centrifuged at 13,000 rpm for 5 min. The gDNA was washed with 70% ethanol, centrifuged at 13,000 rpm for 2 min, and the air-dried pellets were resuspended in 80 µL nuclease-free water supplemented with 0.2 mg/mL RNase (Roth).

For mtDNA quantification, 100 ng DNA was quantified by qPCR amplification of *CgCOX3* as mitochondrial target gene and *CgACT1* as housekeeping gene (primers shown in Table 2).

**TABLE 2:** Primers used for RT-qPCR and mtDNA quantification

Primer	Sequence 5′ to 3′
qPCR *ATG32* fw	ATGGTAAGGCAGAATCACACC
qPCR *ATG32* rev	TTGGTTGTCTTCACGTACTGG
qPCR *ATG11* fw	AGGATGAAAATATGGGGCAGG
qPCR *ATG11* rev	TCAATCCCTTCTTCAGACCCT
qPCR *ATG8* fw	CATTTGAGAAGAGGAAAGCGG
qPCR *ATG8* rev	CATAAGCGATGCAGTTGGTG
qPCR *ATG17* fw	AAGGAAGGTTGTATGCAGGAC
qPCR *ATG17* rev	TCTCCTGGCCATATTGTCTCT
qPCR *PDR1* fw	TGGTCGATGAATTGTTTGGGT
qPCR *PDR1* rev	TGGTGTAGGAGTCATAGGCAT
qPCR *CDR1* fw	GTATTCCGGTTTTGCAATCCC
qPCR *CDR1* rev	CTCAAGAAGTCGTCACCCAAA
qPCR *CDR2* fw	ACCGAAAGAGTATGTGCATCC
qPCR *CDR2* rev	TACCCTCCTTCTTCATACGCT
mtDNA *CgCOX3* fw	TCAAGCAGTACAACCTACAGA
mtDNA *CgCOX3* rev	TGTAAACCAGTACCAGCATAGA
*CgACT1* fw	GGTGACGGCGATTATGAGTTA
*CgACT1* rev	AACACCATTACCGGGCAATAA

### RNA isolation

To investigate the regulation of autophagy and efflux pump genes in the *C. glabrata ksp1*∆ mutants and the *petites*, overnight cultures of *C. glabrata* strains were centrifuged at 3,000 rpm for 2 min. The supernatant was removed, and the pellet was shock-frozen in liquid nitrogen. Thawed pellets were resuspended in 600 µL RNeasy Lysis (RLT) buffer (Qiagen) containing 1% β-mercaptoethanol, and RNA was isolated as described before (80).

The macrophage response to *C. glabrata* and the yeast-locked *C. albicans efg1*ΔΔ/*cph1*ΔΔ mutant was determined in the escape model. In brief, hMDMs were seeded in a six-well plate (1 × 10^6^ hMDMs/well) and infected with an MOI of 1 in RPMI+10% HS. At nine time points (0 h, 3 h, 8 h, 24 h, 32 h, 48 h, 56 h, 72 h, and 80 h), the medium was aspirated, and 500 µL RNeasy Lysis (RLT) buffer (Qiagen) containing 1% β-mercaptoethanol was added. The host cells were detached using a cell scraper and vortexed for 20 s to lyse the cells. To remove the fungal cells, the solution was centrifuged for 10 min at 20,000 × *g* at 4°C. The host RNA-containing supernatant was transferred into an RNase-free tube and frozen in liquid nitrogen. RNA was isolated from the thawed supernatant using the RNeasy Mini Kit (Qiagen) according to the manufacturer’s instructions. RNA concentrations were quantified using a NanoDrop 1000 Spectrophotometer (ThermoFisher Scientific), and RNA quality was assessed with an Agilent 2100 Bioanalyzer (Agilent Technologies).

### Quantitative reverse transcription PCR

Isolated RNA (500 ng) was treated with DNase I (Fermentas) following the manufacturer’s instructions, and subsequently transcribed into cDNA using 0.5 µg pligo(dT)_12–18_ primer, 200 U Superscript III Reverse Transcriptase and 40 U RNaseOUT Recombinant RNase Inhibitor (Thermo Fisher Scientific). The obtained cDNA was diluted 1:5 and used for qPCR with GoTaq qPCR Master Mix (Promega) in a CFX96 thermocycler (Bio-Rad). The expression levels were normalized against *CgACT1*. All primers used are listed in Table 2.

### RNA sequencing and transcriptional analysis

Library preparation and RNA sequencing were carried out at Genewiz/Azenta (Leipzig, Germany). After poly(A) selection, the mRNA was fragmented, and cDNA libraries were generated for each sample. Libraries were sequenced with 2 × 150 read lengths using an Illumina NovaSeq platform.

Reads were aligned to the human standard genome using bowtie2 v2.5.2 (81) with standard settings. Transcripts were counted by Subread featureCounts v2.0.5 (82) followed by median ratio normalization.

Next, we performed gene set enrichment analysis and visualized clustering patterns of our data across different strains and conditions using R (v4.3.2). To visualize the transcriptomic response of the macrophages to both species over time, first principal component analysis was performed using the prcomp function in the stats package (v4.3.2). The first two components were selected, and k-means clustering with two centers was performed using the factoextra package (v1.0.7). Reduced GO term analysis, upregulated and downregulated genes were defined with a log_2_ fold change threshold of ±1.5. Significantly enriched pathways (*P* < 0.05) were identified using the compareCluster function in the ClusterProfiler package (v4.10.1) (83). A subset of pathways of interest was manually curated and visualized.

### Whole blood fungal survival

Human whole blood was freshly drawn from healthy volunteers in anticoagulation tubes with recombinant Hirudin (Sarstedt). *C. glabrata* overnight cultures were prepared as described above, 10^6^ yeasts were added to 490 µL whole blood and incubated at 37°C under gentle rolling of the tubes. Samples were taken after 15 min, 30 min, 60 min, 120 min, and 240 min and plated on YPD in appropriate dilutions in PBS. The plates were incubated at 37°C, and CFUs were determined after 1 day to assess fungal survival and incubated afterward for a further 3 days to check for *petites*.

### Cytokine release quantification

Supernatants of infected primary macrophages (MOI 5) were collected 24 h post infection after centrifuging samples for 10 min at 250 × *g*. The release of IL-8, IL-6, IL-1β, TNFα, and GM-CSF was measured using commercially available human enzyme-linked immunosorbent assay kits (R&D Systems) according to the manufacturer’s protocols.

### Mitophagy staining and heterogeneity measurement

*C. glabrata* overnight cultures were prepared as described above. Subcultures were inoculated 1:100 and grown for 4 h at 37°C. 12 mL of the logarithmically growing culture were harvested and stained with MitoTracker Deep Red FM (Invitrogen) at 37°C for 30 min. After two subsequent wash steps with PBS, the pellet was resuspended in PBS, and 1:100 dilutions were pipetted into 8-chamber ibiTreat µ-slides (ibidi). Images were taken with 40× magnification using water immersion with an Axio Observer Z1 (Zeiss). To determine the population heterogeneity, a region of interest (ROI) of the same size was selected for all images. All fungal cells, *petite* and *grande*, were counted within this ROI. To analyze the mitophagy staining, the images were evaluated using ImageJ 1.51n (National Institute of Health, USA). To avoid a biased selection, at least 15 fungal cells per sample and replicate were selected in the brightfield image. These selected areas were copied to the fluorescence image, and the measured mean gray scale of those was used as “mean fluorescence intensity.”

### TEM of *C. glabrata* cells

For TEM, *C. glabrata* overnight cultures were diluted 1:50 in YPD 1% peptone and incubated for 5 h at 37°C and 180 rpm. After centrifugation, the pellet was washed three times in PBS and adjusted to an OD of 0.5 in 10 mL 0.6 M KCl, 50 mM dithiothreitol, 5 mM EDTA, and 0.5 mg/mL Pronase (Roche). Following an incubation step for 30 min at 30°C and 180 rpm, the fungal suspension was spun down, and the pellet was washed three times in 0.6 M KCl. The pellet was treated with 10 mL of zymolyase buffer (0.6 M KCl, 50 mM dithiothreitol, 5 mM EDTA, 2 mg/mL zymolyase [Seikagaku Biobusiness], pH 7.4) and incubated at 37°C and 180 rpm for 1 h. The fungal suspension was spun down at max. 100 *g* and washed in osmotic medium (1.2 M MgSO_4_, 1 M Na_2_HPO_4_, pH 5.8). The cell-wall-free fungal cells were resuspended in 1 mL fixing agent (4% [wt/vol] formaldehyde and 2.5% [vol/vol] glutaraldehyde in PBS), incubated at room temperature for 2 h, and then kept overnight at 4°C.

For TEM preparation, fixed protoplasts were contrasted with 1% (wt/vol) osmium tetroxide in 100 mM sodium cacodylate buffer (Serva), dehydrated, and infiltrated with araldite resin (Agar Scientific). Ultrathin sections were stained with uranyl acetate and lead citrate, mounted on copper grids, and examined with an EM 900 (Zeiss, Oberkochen, Germany) TEM at 80 kV. Images were acquired using a wide-angle dual-speed 2K CCD camera (Tröndle).

### Statistics and reproducibility

Experiments were performed in at least biological triplicates (*n* = 3) with three independent experiments performed on different days. For experiments using primary macrophages or whole blood, at least six or four different donors were used, respectively. Data were analyzed using GraphPad Prism 10.2.3 (GraphPad Software, La Jolla, CA, USA). Values are presented as mean ± standard deviation (SD) or with individual values for each donor. Statistical tests are indicated in each figure legend. Statistical significance is indicated in the figures as follows: *, *P* ≤ 0.05; **, *P* ≤ 0.01; ***, *P* ≤ 0.001; ****, *P* ≤ 0.0001.

## Data Availability

All sequencing data generated in this study have been deposited in the European Nucleotide Archive (ENA) under accession number PRJEB106507.

## SUPPLEMENTAL MATERIAL

Supplemental Figures mbio.03885-25-s0001.pdf Fig. S1 to S7.

Table S1 mbio.03885-25-s0002.xlsx Primers to generate mutants.

ASM does not own the copyrights to Supplemental Material that may be linked to, or accessed through, an article. The authors have granted ASM a non-exclusive, world-wide license to publish the Supplemental Material files. Please contact the corresponding author directly for reuse.
