# Supplementary material for: “Should I stay or should I go”—a kinase delays escape of Candida glabrata from macrophages
Source: mBio. 2026 Feb 20;17(3):e03885-25. doi: 10.1128/mbio.03885-25 (PMC12977481; doi:10.1128/mbio.03885-25)
Supplement: Supplemental Figures — Fig. S1 to S7. [file mbio.03885-25-s0001.pdf]

Supplementary figures

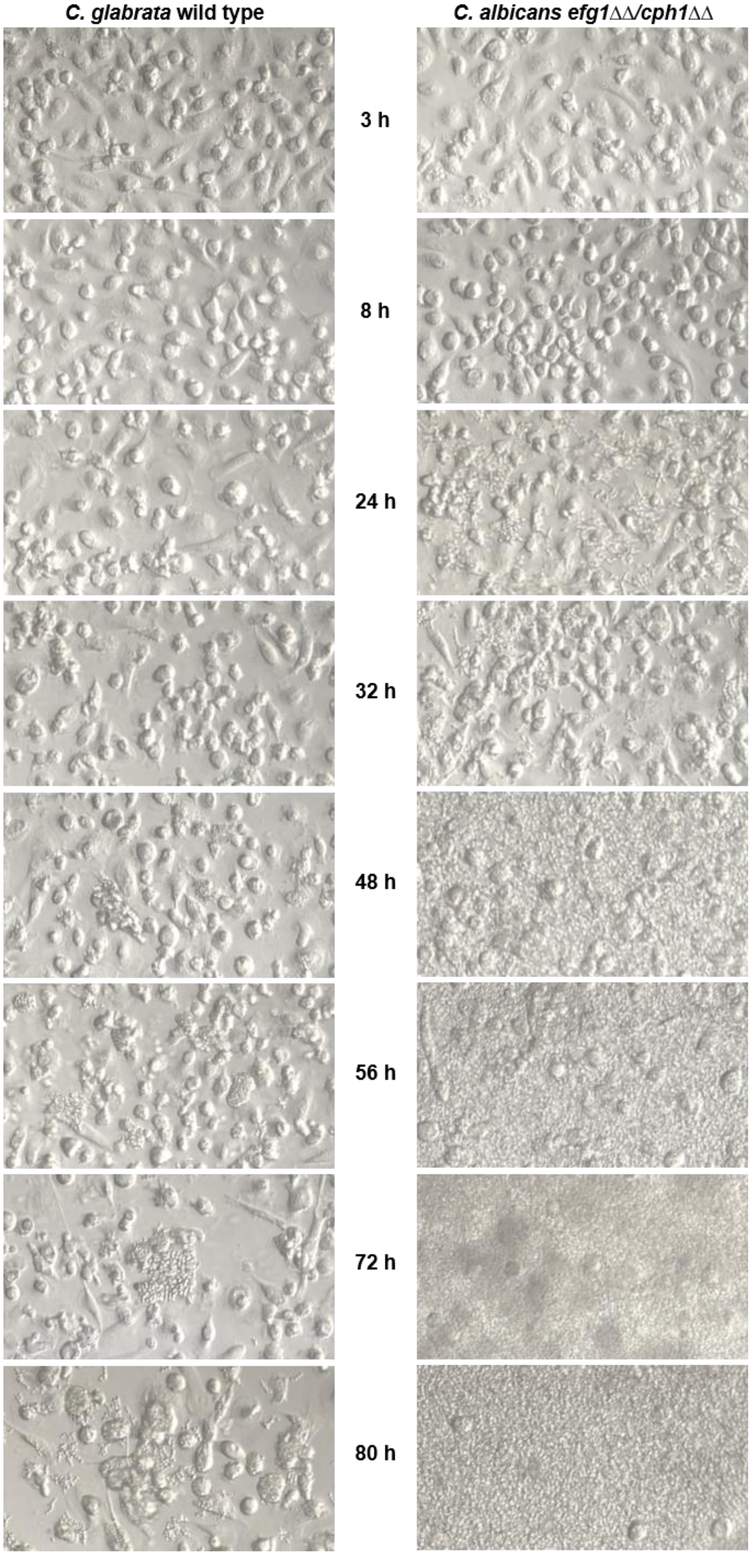

Supplementary figure S1: Representative micrographs of the escape model. Primary human macrophages (hMDMs) were infected with either the *C. glabrata* wild type or the yeast-locked *C. albicans* mutant *efg1*ΔΔ/*cph1*ΔΔ (MOI 1). After 3 h, all non-phagocytosed yeasts were washed away, and the infection process was monitored for 3 days. Images were taken at two time points each day (also chosen for the host transcriptome) with 20× magnification. Representative images are shown for one donor (n = 3 donors).

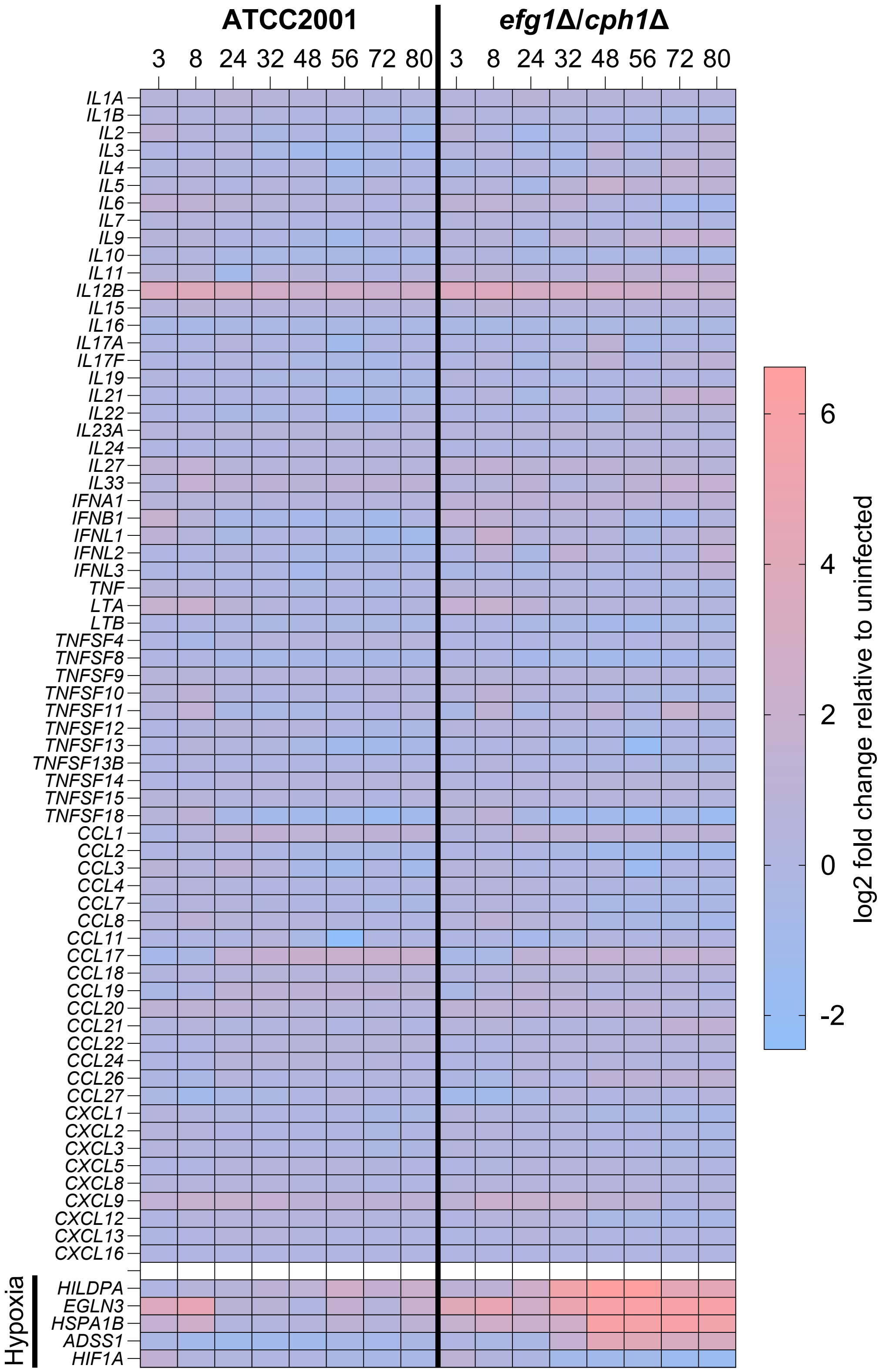

Supplementary figure S2: Differentially regulated macrophage genes. The heatmap shows the log2 fold change of infection-associated genes in ATCC2001- or *efg1*Δ/*cph1*Δ-infected macrophages relative to the uninfected 0 h-time point.

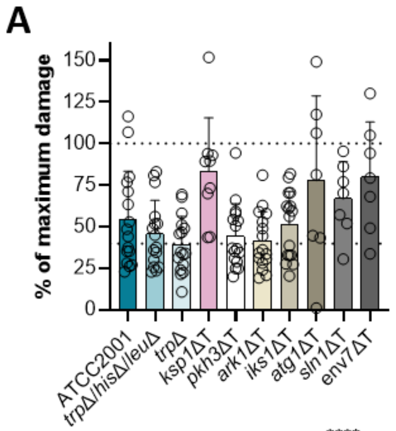

	*ksp1*ΔT	*pkh3*ΔT	*ark1*ΔT	*iks1*ΔT	*atg1*ΔT	*sln1*ΔT	env7ΔT
ATCC2001	*	ns	ns	ns	ns	ns	ns (0.0849)
trpΔ/hisΔ/leuΔ	**	ns	ns	ns	*	ns	*
trpΔ	***	ns	ns	ns	**	ns	**

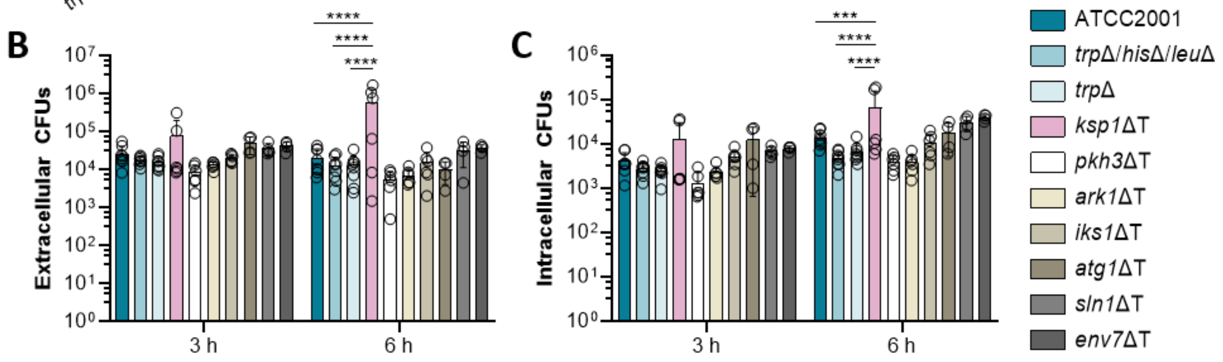

Supplementary figure S3: Characterization of all strongly lysing *C. glabrata* kinase mutants in primary human macrophages. (A) Lactate dehydrogenase release as measure of damage by three parental *C. glabrata* strains and 7 kinase mutants to primary human macrophages 24 h post infection (n = 7-16 donors). The 7 kinase mutants were chosen as all of them showed an enhanced lysis on J774A.1 cells compared to the wild type ATCC2001. Statistical significances among the strains are depicted in the table on the right side and were calculated using a one-way ANOVA with Tukey's multiple comparisons test (*, $p < 0.05$; **, $p < 0.01$; ***, $p < 0.001$). (B, C) Survival of extracellular (B) and intracellular (C) *C. glabrata* parental strains and the kinase mutants within primary human macrophages. For extracellular survival, non-phagocytosed yeast cells were collected and plated at indicated time points. The intracellular CFUs were determined by lysing and plating at indicated time points (n = 4-8 donors). Statistical significance was calculated using a two-way ANOVA with Tukey's multiple comparisons test (***, $p < 0.001$; ****, $p < 0.0001$).

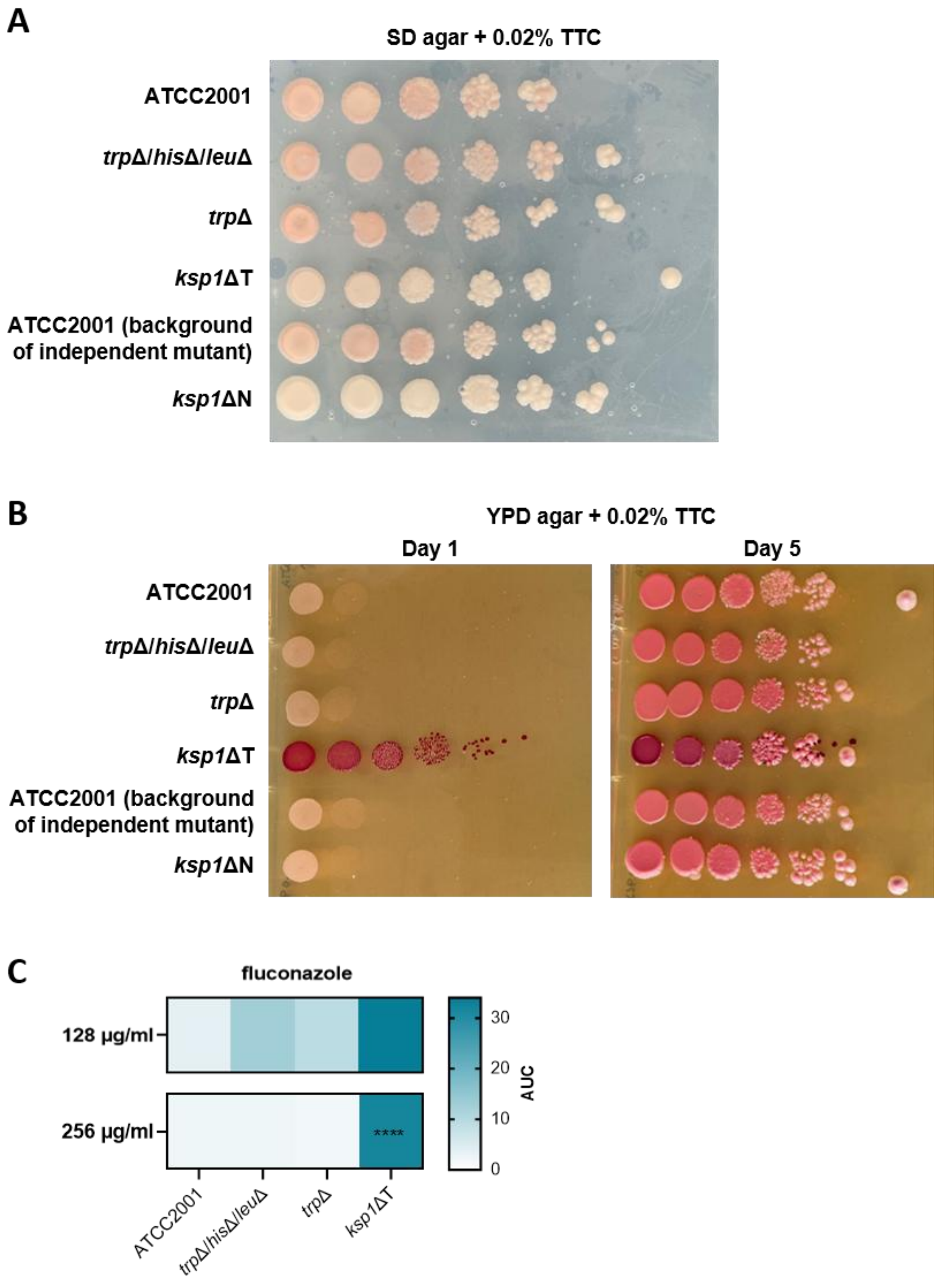

Supplementary figure S4: Further characterization of the potential *ksp1*Δ-derived *petite* phenotype. (A, B) Mitochondrial function was determined by a drop test of serial dilutions of the parental *C. glabrata* strains and *ksp1*Δ kinase mutants on SD+4% glycerol agar (A) or YPD agar (B) containing 0.02% tetrazolium chloride, which indicates functional mitochondria by dye reduction. Plates were grown at 37 °C for up to 4 days (YPD: n = 3, SD: n = 2). (C) Fluconazole susceptibility of the parental *C. glabrata* strains and the *ksp1*Δ kinase mutant measured by performing growth curves in YPD supplemented with the indicated antifungal concentrations at 37 °C for 3 days. Growth is shown as the mean of area under the curve (n = 3). Statistical significance was calculated using a one-way ANOVA with Tukey's

multiple comparisons test (****, $p < 0.0001$).

A

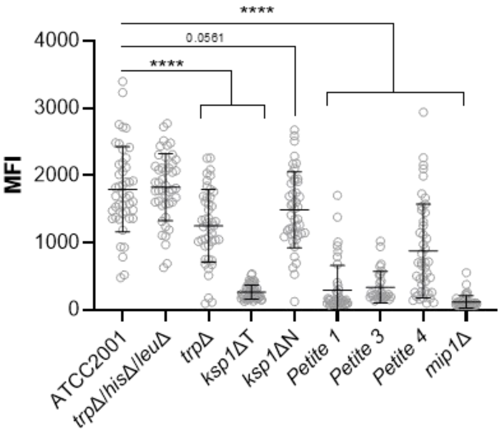
****
0.0561
****
4000
3000
2000
1000
0
MFI
ATCC2001
trpΔ/hisΔ/leuΔ
trpΔ
ksp1ΔT
ksp1ΔN
Petite 1
Petite 3
Petite 4
mip1Δ

B

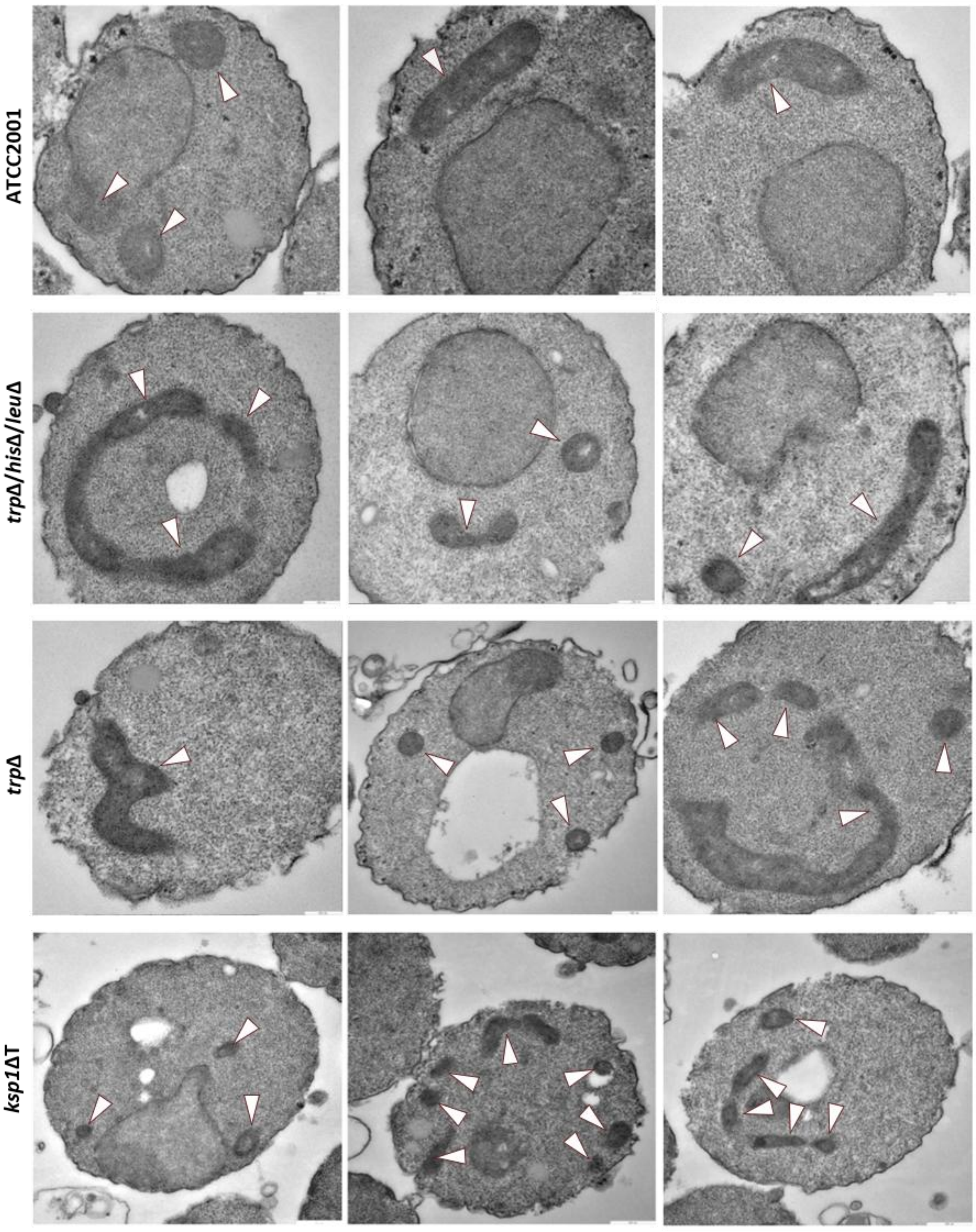
ATCC2001
trpΔ/hisΔ/leuΔ
trpΔ
ksp1ΔT

Supplementary figure S5: MitoTracker staining quantification and representative transmission electron microscopy pictures of the *ksp1*ΔT mutant. (A) Logarithmically grown fungal cells were stained with MitoTracker and the mean fluorescence intensity (MFI) was measured of at least 15 cells per replicate (n = 3). Each dot represents one measured cell. Three *petite* strains as well as the mitochondrial DNA polymerase-deficient *C. glabrata mip1*Δ mutant were included as a MitoTracker-negative control. Statistical significance of comparisons with ATCC2001 were determined using a one-way ANOVA with Tukey's multiple comparisons test (****, p < 0.0001). (B) Pictures were taken from synchronized cultures of the parental *C. glabrata* strains and *ksp1*ΔT kinase mutant. White arrows indicate mitochondria. The scale bar indicates 250 nm.

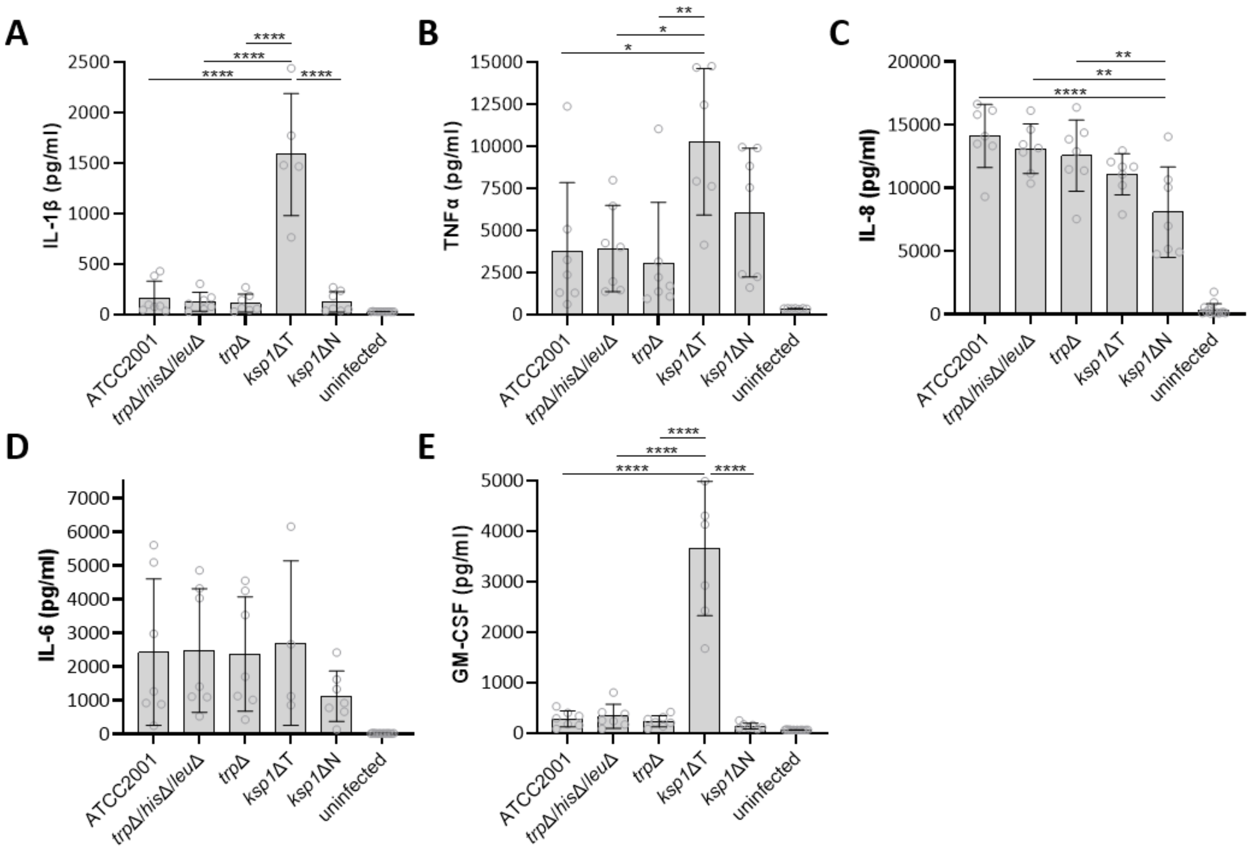

Supplementary figure S6: Immune activation induced by the *ksp1*Δ mutants. Concentration of the cytokines (A) IL-1β, (B) TNFα, (C) IL-8, (D) IL-6, and (E) GM-CSF was determined in supernatants of infected primary macrophages 24 h post infection (n = 7 donors). Statistical significances of comparisons with ATCC2001 were determined using a one-way ANOVA with Tukey's multiple comparisons test (*, p < 0.05; ****, p < 0.0001).

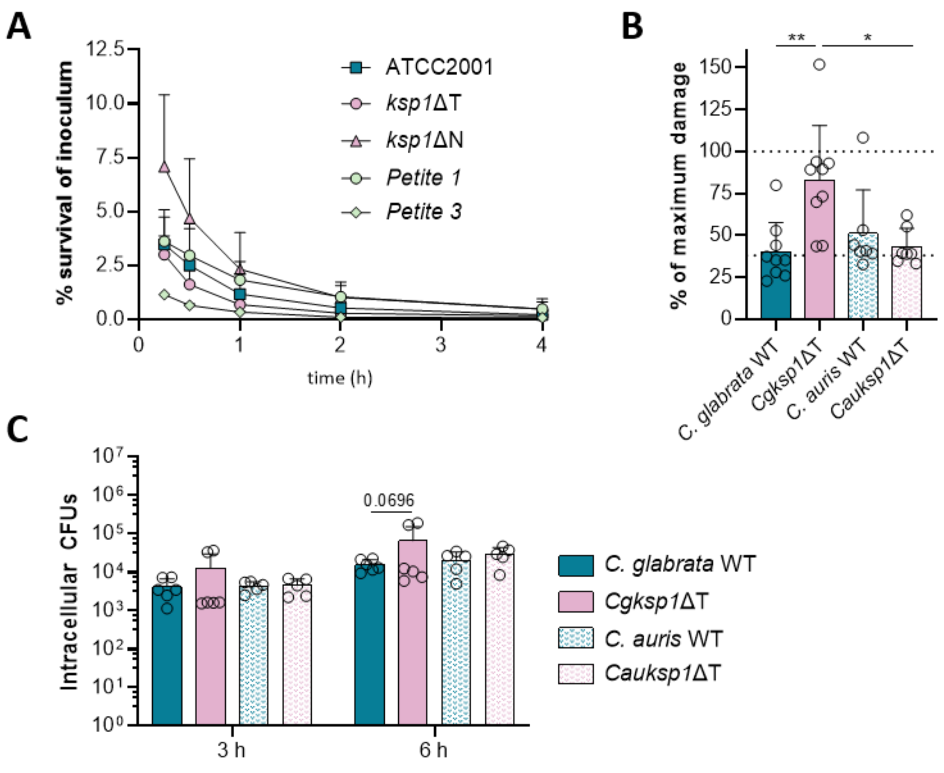

Supplementary figure S7: Survival of the isolated *petites* in whole blood and the role of Ksp1 in the *C. auris*-macrophage interaction. (A) Survival of two *petites* in comparison with the *C. glabrata* wild type and the *ksp1*Δ kinase mutants in whole human blood. Surviving CFUs were determined over the course of 4 h by plating and shown relative to the inoculum (n = 4 donors). (B) Damage induction measured by quantification of lactate dehydrogenase release of the *C. auris* wild type and the *C. auris ksp1*ΔT kinase in primary human macrophages 24 h post infection (7 donors). Lower dashed line indicates the LDH release background measured in an uninfected control. The LDH data for the two *C. glabrata* strains is also depicted in Figure 3. Statistical significance was calculated using a one-way ANOVA with Tukey's multiple comparisons test (*, $p < 0.05$; **, $p < 0.01$). (C) Survival of the intracellular *C. auris* wild type and the *ksp1*ΔT kinase mutant in primary human macrophages determined by lysing and plating intracellular CFUs at the indicated time points (5 donors). The survival data for the two *C. glabrata* strains is also depicted in Figure 3. Statistical significance was calculated using a two-way ANOVA with Tukey's multiple comparisons test.
